# Systematic review and meta-analysis of interventions to improve outcomes for parents or carers of children with anxiety and/or depression

**DOI:** 10.1136/bmjment-2024-301218

**Published:** 2024-09-25

**Authors:** Anthony Tsang, Dania Dahmash, Gretchen Bjornstad, Nikki Rutter, Aleem Nisar, Francesca Horne, Faith Martin

**Affiliations:** 1 Division of Psychology & Mental Health, School of Health Sciences, The University of Manchester, Manchester, UK; 2 Coventry University, Coventry, UK; 3 The Medical School, University of Exeter, Exeter, UK; 4 Applied Research Collaboration South West Peninsula (PenARC), NIHR, Exeter, UK; 5 Department of Sociology, Durham University, Durham, UK; 6 Independent Researcher, UK; 7 Department of Social Work, Care and Communities, Nottingham Trent University, Nottingham, UK; 8 School of Psychology, Cardiff University, Cardiff, UK

**Keywords:** Depression, Anxiety disorders, Child & adolescent psychiatry, Adult psychiatry

## Abstract

**Question:**

Depression and anxiety are common among children and young people and can impact on the well-being of their parents/carers. Dominant intervention approaches include parent training; however, this approach does not directly address parents’ well-being. Our objective was to examine the effect of interventions, with at least a component to directly address the parents’ own well-being, on parents’ well-being outcomes, including stress, depression and anxiety.

**Study selection and analysis:**

A systematic search was performed in the following: MEDLINE, EMBASE, CINAHL, AMED, PsycINFO, Scopus, CENTRAL, Web of Science Core Collection (six citation indexes) and WHO ICTRP from inception to 30 December 2023. Interventions that aimed to support parents/carers managing the impact of their child’s/young person’s mental health were eligible. EPHPP (Effective Public Health Practice Project) was used to quality appraise the included studies. A meta-analysis of relevant outcomes was conducted.

**Findings:**

Fifteen studies were eligible comprising 812 parents/carers. Global methodological quality varied. Seven outcomes (anxiety, depression, stress, burden, self-efficacy, quality of life and knowledge of mood disorders) were synthesised at post-intervention. A small reduction in parental/carer anxiety favouring intervention was indicated in one of the analyses (*g*=−0.26, 95% CI −0.44 to –0.09, p=0.02), when excluding an influential case. Three outcomes were synthesised at follow-up, none of which were statistically significant.

**Conclusions:**

Interventions directly addressing the well-being for parents of children with anxiety and/or depression appear not to be effective overall. Clearer conceptualisation of factors linked to parental distress is required to create more targeted interventions.

**PROSPERO registration number:**

CRD42022344453.

**WHAT IS ALREADY KNOWN ON THIS TOPIC**

- There is a link between child and young people (CYP) and parent mental health, with interventions working with parents to improve child outcomes.
- There is limited research on interventions aimed specifically at supporting parents of CYP with anxiety and/or depression.

**WHAT THIS STUDY ADDS**

- We identified 15 studies reporting outcomes for parents for interventions, where the intervention included content directly aiming to improve parents’ well-being. Overall, these were not found to be effective.
- The interventions were diverse in their content and lacked clear conceptualisations of factors linked to parents’ distress.

**HOW THIS STUDY MIGHT AFFECT RESEARCH, PRACTICE OR POLICY**

- The effectiveness of direct support for parents’ well-being where their CYP has anxiety and/or depression requires further investigation, with fully powered, randomised controlled trials, with clearly specified proposed mechanisms.
- The relative importance of increasing parenting self-efficacy via parent training versus supporting parents’ own psychological well-being directly remains unclear and warrants further investigation.

## Background

 Anxiety and depressive disorders are the most commonly occurring mental health conditions among children and young people (CYP).^1^ In the UK, prior to the COVID-19 pandemic, 1.25 million young people aged 5–19 years had a diagnosable mental illness, but the Child and Adolescent Mental Health Services had the capacity to see just one-third of these.^2^ The prevalence of depression and anxiety symptoms have doubled since the pandemic globally,^3^ placing further pressure on services with more families affected by mental health difficulties in their CYP.

Given the high prevalence of anxiety and/or depression in childhood, a large number of parents and carers (hereafter ‘parents’) are often supporting their CYP without professional input. In England, around just one-third of young people with mental health problems access formal mental health services, which have long waiting lists.^4^ The time until diagnosis, and effective intervention if offered, may be lengthy. Parents encounter uncertainty as to who can assist their CYP, creating additional demands for parental support.^5^

CYP’s mental health is closely linked to that of their parents.^6 7^ Research has extensively investigated the impact of parental mental health on CYP. However, there is less focus on the impact of a child’s mental health on the parents’ own mental health and interventions to support parents. It is known that CYP’s mental health difficulties can impact parents, leading to depression, stress, self-blame and feelings of helplessness, affecting work attendance and relationship with their CYP.^8^,^17^

Despite the high prevalence of CYP experiencing anxiety and/or depression and its impact on parents, there is limited work developing interventions to support their parents. Current interventions often prioritise using parents as a conduit to improve CYP outcomes. Examples include parents being trained to deliver interventions directly to their children^18^ and improve parenting to reduce CYP distress via parent training.^19^ The mechanism to improve parental well-being in these interventions is typically indirect and does not address the impact of CYP’s mental health difficulties on the parent.^20 21^ The interventions (or elements of interventions) that aim to specifically, directly support parents have not yet been systematically identified or summarised. A systematic review and meta-analysis may elucidate the interventions necessary to improve parents’ well-being and facilitate a fine-grained evaluation of their effectiveness.

### Objective

This review focuses on identifying, summarising and evaluating the effectiveness of interventions that support parents of CYP with anxiety and/or depression.

## Methods

### Registration and deviations from protocol

This review followed the PRISMA (Preferred Reporting Items for Systematic Reviews and Meta-Analyses) 2020 reporting guidelines.^22^ A detailed protocol was published^23^ and was registered in PROSPERO (number: CRD42022344453).^24^ This manuscript focuses on experimental studies with outcomes; another manuscript detailing all relevant interventions is being prepared. We originally planned separate reviews based on whether the child received professional health services. However, this proved infeasible during searching. Planned subgroup analyses to examine this were also not possible due to lack of reporting. We added Web of Science Core Collection and AMED to our search, based on advice from an information specialist. Cochrane Risk of Bias V.2 was replaced with the Effective Public Health Practice Project (EPHPP) tool due to the wide-ranging experimental designs.

#### Patient and public involvement (PPI)

The systematic review was conducted with the involvement of parents of young people who have or have had depression and/or anxiety. Our research team included parents with lived experience. Parents (two mothers, two fathers) attended a planning workshop during the development of the aims and scope. They described a perception that despite parent support groups being common in practice, they were curious about research examining their impact. They shared views that research examining parenting interventions focus on ‘how to parent’ but less on interventions that focus on ‘how the parent is’. This led to the final research objectives, criteria for eligibility for the interventions and specification of the outcomes of interest as relating to parent’s well-being.

#### Eligibility criteria, search strategy and selection process

The eligibility criteria outlined in table 1 were developed using the PICOS framework.^25^ A combination of subject headings and free-text terms was used for five search concepts that pertained to (1) CYP, (2) mental health conditions, (3) parents/carers, (4) intervention and (5) outcomes. A comprehensive search was conducted (FM) using the following information sources: PsycInfo (EBSCO), CINAHL (EBSCO), AMED (EBSCO), MEDLINE (EBSCO), EMBASE (Ovid), Scopus, CENTRAL, WHO ICTRP and Web of Science Core Collection (SCI-E, SSCI, CPCI-S, A&HCI, CPCI-SSH and ESCI). Searches were conducted from inception of database/trial registry to 30 December 2023, limited by English language (online supplemental material 1). To reduce the file drawer effect, backward and forward citation searches were performed (FH) on all eligible studies using CitationChaser.^26^

**Table 1:** Eligibility criteria

PICOS	Inclusion criteria	Exclusion criteria
Population	Study participants must have been parents or carers (any adult in a parenting role, including foster carers and adoptive parents) of CYP, where at least 70% of the CYP were aged 5–18 years with an anxiety and/or depression disorder. The term CYP was used here as our PPI group recommended this term, owing to their view that teenagers may find the term children applicable to them. The decision for the age range was due to that in many contexts, child mental health services serve between the ages of 5 and 18.^55^ The CYP must have had a clinical level of anxiety and/or depression, as measured by a validated measure or based on medical diagnosis (eg, young people have been assessed by general practitioner and referred to specialist mental health services with anxiety and/or depression). Anxiety disorders, including generalised anxiety, obsessive compulsive disorder and social phobia, were included. Depressive disorders at any clinical level were included.	PTSD was excluded as the experience of the traumatic event may have caused trauma in the parent and PTSD is no longer considered an anxiety disorder.^56^ Bipolar disorders were excluded based on the clinical classifications of these difficulties.^57^ Procedural anxiety (eg, deriving from a medical paediatric setting) If the primary diagnosis for inclusion in the study is related to intellectual disability, neurodiversity, attention deficit diagnoses or physical problems. This decision was based on that it is not possible to separate the impact of other diagnoses on the parents from the impact of their young person having anxiety and/or depression.
Intervention	Interventions must include at least one component that directly seeks to support the parent with the impact of having a young person with anxiety and/or depression. Educational interventions were included where the education also extends to discussing the impact on parents and how they can manage this.	The following interventions were excluded: focused on primary prevention, only offered parent training, trained the parent to deliver therapy to the young person or only provided education about anxiety/depression as they do not focus on supporting the parent with their distress.
Comparison	Studies with or without a comparison group were included	No exclusion criteria
Outcomes	Outcomes that focused on the parents/carers that included but not limited to the following: parental mental health, mental well-being, stress, burden, burnout or satisfaction. All relevant outcomes must have been measured with a validated measure. For example, Parent Health Questionnaire-9^58^ to measure depression.	Outcomes that solely focused on the young person.
Study type	Both peer-reviewed and non-peer-reviewed evidence sources included the following quantitative and mixed-method research designs: randomised controlled trials, quasi-experimental designs and controlled trials.	Non-experimental and qualitative study designs.

CYP, children and young people; PPI, patient and public involvement; PTSD, Post-traumatic stress disorder.

EndNote (V.X8) was used to deduplicate all imported search records using the in-built function. Remaining records were subsequently imported to Rayyan^27^ to facilitate a two-step blind screening approach. Title and abstracts of all records were screened by two independent screeners followed by full-text reports (AT, DD, NR and FH). Two screeners (AT and FH) independently reviewed all supplementary search results. All conflicts were arbitrated by a third screener (FM).

#### Data extraction and quality assessment

Data were extracted by two reviewers (AT and DD) and verified for accuracy by another reviewer (NR or FM). Four categories of data related to the following characteristics were extracted: study (eg, author and study design), population (eg, CYP age), intervention (eg, name and summary) and outcome (eg, measures used).

The EPHPP tool for quantitative studies was used to evaluate methodological quality due to multiple study designs. This was performed by two independent reviewers (AT, DD, FH and FM) and discrepancies were resolved by a third independent reviewer or via discussion (FM or AT). EPHPP is composed of six components (ie, selection bias, study design, confounders, blinding, data collection method, and withdrawals and dropouts) that were rated as either weak, moderate or strong. A global rating is considered weak if multiple components were rated as weak, moderate if only one component was rated as weak and strong if no individual component rated as weak.^28^

### Data analysis and synthesis

A preliminary synthesis of studies was conducted by tabulation, clustering based on characteristics and content analysis of intervention components, and vote-counting was used as a descriptive tool to provide frequencies of extracted data.^29^ The transformation of statistics into a common metric was performed for outcomes with only one effect. Statistics that were incompatible for transformation were reported separately.

#### Effect size computation and integration method

All analyses were carried out using R (V.4.3.1). Two main approaches were used for effect size computation of continuous parental outcomes at post-intervention and follow-up. Precalculated effect sizes were used when raw data are absent and in analyses that considered both between-group and within-group studies. Uncalculated effect sizes were used if raw data were provided. Cohen’s *d* was the selected main effect for analyses considering both between-group and within-group studies using precalculated effects. In the absence of descriptive statistics, other computational methods were used. Cohen’s *d*_av was calculated for within-group studies. This accounted for the effect of different experimental designs and allowed for a more direct comparison with classical Cohen’s *d* often used for between-group designs.^30 31^ Hedges’ *g* were calculated to correct for small sample bias that considered between-group studies.^32^ Effect size interpretation followed Cohen’s (1988) convention of small (0.2), medium (0.5) and large (0.8).^33^

The following steps ensured consistency and comparability across studies and avoided dependency of effects: (1) raw data were used over reported effects by authors to ensure a consistent approach, (2) reported SEs were first converted into SD to adhere to the same approach of computation of effects, (3) studies that reported data for multiple follow-up timepoints were combined by calculating a mean difference between the timepoints, (4) statistics deriving from intention-to-treat analyses were selected over per-protocol analyses, (5) pre-post effects were avoided in between-group studies by calculating effects between intervention and control groups at post-intervention and follow-up separately to discern the effects of the intervention by controlling for natural processes,^34^ (6) the parent-only group was considered the intervention group to better isolate the effect on parental outcomes in studies with multiple treatment arms, and (7) composite outcome scores were chosen over subscales to reduce multiple data points.

The inverse variance method using a random-effects model was used to pool effect sizes due to anticipated high levels of heterogeneity. The restricted maximum likelihood estimator was used to calculate the variance of between-study heterogeneity as recommended for continuous data.^35^ To assess and quantify the total variability due to between-study heterogeneity, τ^2^ and *I*^2^ were reported, respectively. The thresholds of interpretation of *I*^2^ was based on Higgin’s *et al*^36^ and were as follows: low (25%), moderate (50%), substantial (75%). 95% prediction intervals (PIs) were calculated to provide the dispersion of the true effect sizes of future studies. Outlier and influence analyses were performed to assess the robustness of summary effects. Where outliers or influential studies were identified, meta-analyses were re-ran without such cases. Publication bias was examined through visual inspection of funnel plots and quantified via Egger’s test.^37^ Sensitivity analyses were conducted, which excluded either between-group or within-group studies. All statistical data, functions, packages used and coding are available in the online supplemental R Markdown file.

## Findings

### Study selection

**Figure 1:**
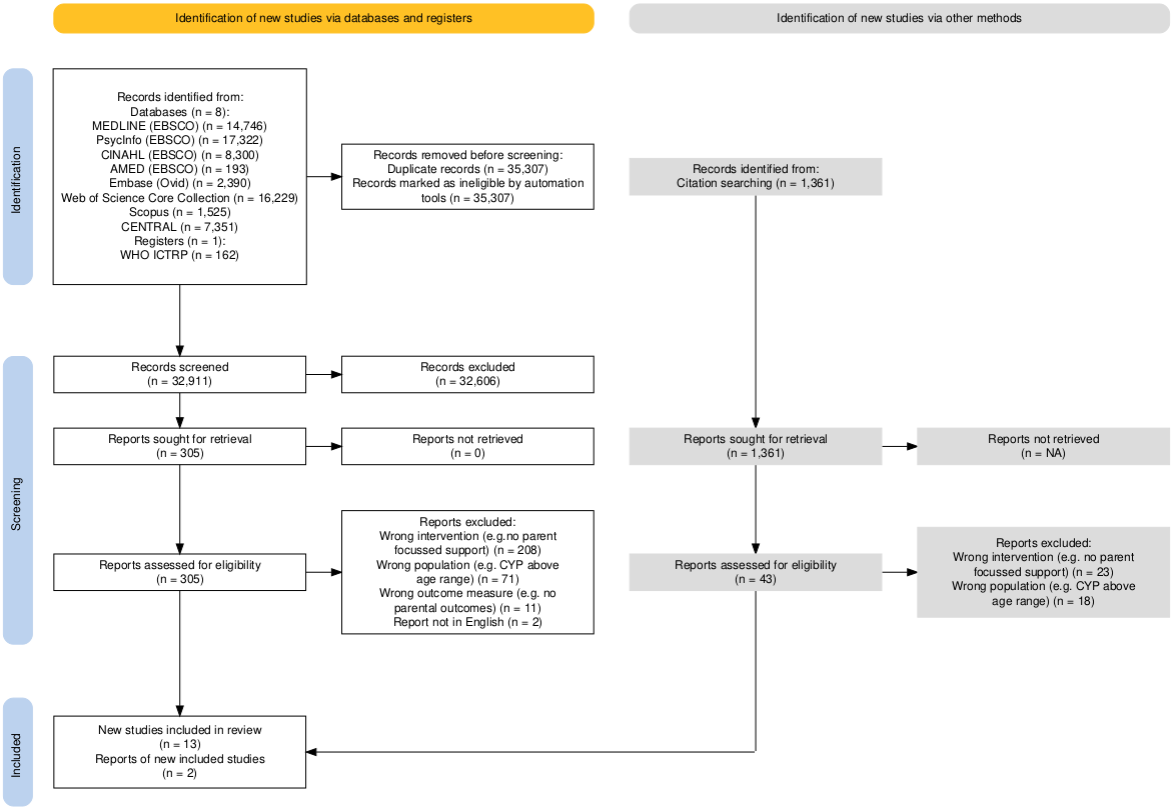
PRISMA (Preferred Reporting Items for Systematic Reviews and Meta-Analyses) 2020 flow diagram of searches. CYP, child and young people.

The systematic electronic searches yielded a total of 32 911 unique records. Backward citation searching and forward citation searching found 682 and 679 records, respectively. A total of 348 full-text reports were screened, yielding 15 included studies. A summary of the study selection process was illustrated in figure 1.

### Study, participant and intervention characteristics

An overview and summary of the characteristics of studies can be found in online supplemental material 2. There were five study designs, with randomised controlled trial^38^,^46^ being the most prevalent.

Two studies were unpublished.^47 48^ There was a global representation with studies covering an 18-year period (ie, 2003–2022).

A total of 812 parents/carers were included, with an age range between 19 and 78 years. CYP age range was between 6 and 24 years, with a mean age range of 6.68–16.83. Mothers constituted the majority of participants, ranging from 68%^39^ of the sample up to 100%.^38^ Non-biological parents^43 49^ and grandparents^43^ were participants in 20% of studies.

Psychoeducation was the most prevalent component within interventions featured in 12 studies.^3940 42^,^47 49^ Many studies included aspects of cognitive behavioural therapy, family therapy, coping and communication strategies. Unique intervention typologies included mindfulness,^50^ attachment focused strategies^51^ and social therapy.^42^ The most common mode of delivery was face-to-face, except in three studies that used an online platform,^42^ web-based and teleconferences,^43^ and a combination of telephone and face-to-face.^41^ The frequency of sessions differed considerably, ranging from 3^44^ to 157 (*m*=14.2, *SD*=9.3) sessions.^48^ The length of interventions ranged from 4 weeks^38^ to 6 months^48^; however, this detail was rarely reported in the studies.

### Methodological quality

The studies presented varied levels of overall global methodological quality. Fifty-six per cent of studies were globally rated as moderate quality, 25% as strong quality and 19% as weak quality. All studies were rated as strong for the components ‘confounders’ and ‘data collection methods’. Additionally, 69% and 63% of studies were rated as strong for ‘study design’ and ‘withdrawal and drop-outs’, respectively. The most prevalent methodological issues were ‘selection bias’ and ‘blinding’. Further, 93% of studies were rated as moderate quality for selection bias, suggesting the selected participants may not have been very representative of the target population. Most studies (69%) were considered weak for ‘blinding’ as measurement of outcomes were largely self-reported, increasing potential reporting bias. Methodological quality appraisals for each study are summarised in online supplemental material 3.

### Parental outcomes at post-intervention

Forty-one effects were identified for seven outcomes at post-intervention. The majority of the analyses (*k*=32) revealed small summary effects with Cohen’s *d* or Hedges’ *g* ranging from −0.41 to 0.41. Summary effects for anxiety (*k*=9; *n*=220), stress (*k*=8; *n*=187), depression (*k*=10; *n*=247) and burden (*k*=5; *n*=273) were not statistically significant. Similarly, knowledge of mood disorders (*d*=0.80; k=2; *n*=58), self-efficacy (*d=*0.69; *k*=3; *n*=128) and quality of life (*d*=0.67; *k*=2; *n*=65) demonstrated no evidence of difference as their respective 95% CIs included zero.

The between-study heterogeneity variance was low for analyses concerning anxiety, knowledge of mood disorders, stress, and self-efficacy (within-group) and burden (within-group) and with an *I*^2^ range of 0% to 48.3%. Substantial levels of between-study heterogeneity with an *I*^2^ range of 82.4% to 94% and τ^2^ range of 0.56 to 1.33 was found for self-efficacy, quality of life and burden. The wide 95% PIs for all analyses indicated negative interventional effects cannot be dismissed in future studies.

No outliers were detected in the analyses. However, influential studies were identified in four analyses. One-study-removal analysis indicated that one study^46^ had an undue influence on anxiety (between-group). The magnitude of this effect was significant, *g*=−0.26 (95% CI −0.44 to –0.09, p=0.02) with its exclusion. Salari *et al*^18^ exerted undue influence on the levels of heterogeneity in depression. Removing this study indicated statistical homogeneity, τ^2^=0.03 (95% CI 0.00, 0.81), *I*^2^=37.4%. The narrower PI confirmed this homogeneity (95%–0.63%, 0.82). This pattern was present in the sensitivity analysis of depression (between-group only). Excluding two studies^18^ ^45^ reduced heterogeneity levels to low, τ^2^=0.02, *I*^2^=16.1%. Statistical homogeneity was also observed in the analysis for stress when Gleeson *et al*^42^ was excluded (*I*^2^=7.4%). However, the PI remained similarly broad, suggesting that negative intervention effects cannot be ruled out in future studies.

Publication bias was not observed in any of the analyses. The results for each outcome can be found in table 2.

**Table 2:** Post-intervention and follow-up effects for parental/carer outcomes

Parental outcome	Post-treatment effect	Heterogeneity statistics	Outlier detection (Y/N)	Presence of influential studies (Y/N)	Egger’s test	Quality based on EPHPP
**Post-treatment effects**						
Anxiety (n=5)^39 42 45 46^	*d*=−0.11 (95% CI −0.35, 0.13, p=0.27)	τ^2^=0 (95% CI 0.00, 0.28), *I*^2^=0%, 95% PI=−0.50, 0.27	N	N	β=−0.86 (95% CI −3.88, 2.15, p=0.61)	Strong^45 46^ Moderate^39^ Weak^42^
Anxiety between-group only (n=4)^39 45 46^	*g*=−0.14 (95% CI −0.52, 0.24, p=0.33)	τ^2^=0.00 (95% CI 0.00, 0.70, *I*^2^=0%, 95% PI=−0.79, 0.51	N	Y	β=−0.93 (95% CI −7.09, 5.32, p=0.80)	Strong^45 46^ Moderate^39^ Weak
Depression (n=6)^41 42 45 46 50^	*d*=0.22 (95% −0.24, 0.68, p=0.28)	τ^2^=0.10 (0.00, 1.21), *I*^2^=59.4%, 95% PI=−0.79, 1.23	N	Y	β=6.06 (95% −10.46, 2.69, p=0.52)	Strong^45 46^ Moderate^41 50^ Weak^42^
Depression between-group only (n=4)^41 45 46^	*g*=0.41 (95% CI −0.28, 1.10, p=0.16)	τ^2^=0.10 (95% CI 0.00, 2.70), *I*^2^=54%, 95% PI=−1.23, 2.05	N	Y	β=8.56 (95% CI 1.05, 16.05, p=0.16)	Strong^45 46^ Moderate^41^ Weak
Stress (n=5)^42 45 46 51^	*d*=−0.04 (95% CI −0.46, 0.38, p=0.80)	τ^2^=0.05 (95% CI 0.00, 0.88), *I*^2^=39.5%, 95% PI=−0.89, 0.81	N	Y	β=5.05 (95% CI −0.37, 10.47, p=0.17)	Strong^45 46^ Moderate^51^ Weak^42^
Stress between-group only (n=3)^45 46^	*d*=0.23 (95% CI −0.04, 0.49, p=0.07)	τ^2^=0.00 (0.00, 0.47), *I*^2^=0%, 95% PI −1.88, 2.33	N/A	N/A	N/A	Strong^45 46^ Weak
Burden (n=3)^41 43 48^	*d*=0.04 (95% CI −1.94, 2.01, p=0.94)	τ^2^=0.57 (0.12, 25.86), *I*^2^=91.9%, 95% PI=−11.16, 11.23	N/A	N/A	N/A	Moderate^41 43 48^
Burden within-group only (n=2)^43 48^	*d_*av=−0.41 (95% CI −1.78, 0.96, p=0.16)	τ^2^=0.01, *I*^2^=48.3%	N/A	N/A	N/A	Moderate^43 48^
Self-efficacy (n=3)^43 46 51^	*d*=0.69 (95% CI −0.72, 2.10, p=0.17)	τ^2^=0.26, *I*^2^=82.4%, 95% PI=−7.10, 8.45	N/A	N/A	N/A	Strong^46^ Moderate^43 51^
Self-efficacy within-group only (n=2)^43 51^	*d_*av=1.05 (95% CI −1.02, 3.13, p=0.10)	τ^2^=0.02, *I*^2^=15.6%	N/A	N/A	N/A	Moderate^43 51^
Quality of life (n=2)^38 42^	*d*=0.67 (95% CI −9.99, 11.34, p=0.57)	τ^2^=1.33, *I*^2^=94%	N/A	N/A	N/A	Moderate^38^ Weak^42^
Knowledge of mood disorders (n=2)^40 49^	*d*=0.80 (95% CI −2.04, 3.64, p=0.17)	τ^2^=0.02, *I*^2^=17.6%	N/A	N/A	N/A	Moderate^40 49^
**Follow-up effects**						
Anxiety (n=3)^39 44 45^	*d*=−0.56 (95% CI −1.46, 0.34, p=0.12)	τ^2^=<0.001 (95% CI 0.00, 14.14), *I*^2^=0%, 95% PI=−3.43, 2.31	N/A	N/A	N/A	Strong^44 45^ Moderate^39^
Depression (n=3)^41 45 48^	*d*=0.08 (95% CI −1.43, 1.59, p=0.85)	τ^2^=0.30 (95% CI 0.03, 15.58), *I*^2^=81.7%, 95% PI=8.08, 8.24	N/A	N/A	N/A	Strong^45^ Moderate^41 48^
Depression between-group only (n=2)^41 45^	*g*=0.18 (95% −7.57, 7.93, p=0.82)	τ^2^=0.68, *I*^2^=90.2%	N/A	N/A	N/A	Strong^45^ Moderate^41^
Knowledge of mood disorders (n=2)^40 49^	*d*=0.30 (95% CI −2.12, 2.72, p=0.36)	τ^2^=0.00, *I*^2^=0%	N/A	N/A	N/A	Moderate^40 49^

EPHPP, Effective Public Health Practice Project; N, no; N/A, not applicable; Y, yes.

### Parental outcomes at follow-up

Ten effects were found at follow-up with small summary effects for depression (*k*=5; *n*=269) and knowledge of mood disorders (*k*=2; *n*=113) that ranged from *d*/*g*=0.08 to 0.30. A medium-sized magnitude of effect was observed for anxiety (*k*=3; *n*=102), *d*=−0.56 (95% CI −1.46, 0.34, p=0.12). None of these effects were statistically significant. Substantial between-study heterogeneity was observed in all analyses that ranged from *I*^2^=90.2% to 95%, except for knowledge of mood disorders (*I*^2^=0%). All analyses including forest and funnel plots can be found in the online supplemental R Markdown file.

### Outcomes with individual effects

There were 21 unique reported parental outcomes (30 total effects) across 11 studies. Racey *et al*^50^ reported a total of four outcomes at post-intervention, of which two were statistically significant. This included greater decentring (*d*_av=0.88, 95% CI 0.31, 1.43) and reduction in rumination (*d*_av=−0.56, 95% CI −1.03 to –0.08) both favouring the intervention. Bertino *et al*^39^ reported four outcomes at post-intervention and follow-up at 6 months, none of which were statistically significant. This trend applied to outcomes reported in the studies Gerkensmeyer *et al*^41^ and Fristad *et al*.^40^ Registad *et al*^51^ measured stress at follow-up, which demonstrated a non-significant effect and adolescent-parent relationship at post-intervention and follow-up, with the former showing a significant difference favouring the intervention group (*d*_av=−0.63, 95% CI −1.18 to –0.07).

Treatment beliefs was the only outcome reported in MacPherson *et al*,^49^ which was measured at three separate time points (post-intervention, 6-month and 12-month follow-up). The effect was only significant at post-intervention (*d*_av=0.44, 95% CI 0.04, 0.84), with greater treatment beliefs favouring intervention. Significant improvements for parents/carers were found in Khor *et al*^43^ at post-intervention pertaining to parent-adolescent attachment (*d*_av=0.45, 95% CI 0.19, 0.71) and parental behaviours associated with reducing anxiety/depression in adolescents (*d*_av=0.76, 95% CI 0.48, 1.03). Global functioning at post-intervention reported by Salari *et al*^18^ showed a significant moderate effect favouring intervention, *g*=0.71, 95% CI −0.01, 1.41. Pina^47^ reported a significant main effect of time on parental anxiety at both post-treatment (*F*(1,66), 16.89, p<0.001; *η*^2^=0.20) and follow-up (*F*(1,41) = 5.73, p<0.05, *η*^2^=0.1). All effects and data can be found in the online supplemental R Markdown file.

## Conclusions and clinical implications

This review analysed interventions directly addressing parents’ needs with managing their CYP’s anxiety and/or depression, synthesising seven outcomes post-intervention and three at follow-up. A reduction in parental/carer anxiety at post-intervention favouring intervention (between-group study designs) with the exclusion of an influential case was the only significant effect. Between-study heterogeneity was low for majority of the analyses. Publication bias was absent throughout all the analyses.

The overall meta-analytical results indicated that the included interventions that have a component specifically focused on parents’ well-being may not be effective at improving well-being for parents of CYP with anxiety and/or depression on parents/carers. Several factors are key to understanding this finding. Extending Lawrence *et al*’s^8^ observation of significant variation in focus, extent and logic of parental involvement to treat CYP themselves, there is similarly a lack of specificity in what elements of parental well-being interventions seek to improve, and through which mechanisms. For instance, interventions may measure parental distress without indicating which components address which mechanisms to alleviate distress. There is ambiguity about whether stress reduction results from alleviating CYP distress in joint CYP-parental interventions or from enhancing parenting skills, which is expected to reduce parental distress through increased self-efficacy. It remains unclear if it is useful to directly seek to improve parents’ welling, or whether this is only possible via the improvement of CYP outcomes. For some parents, pre-existing mental health difficulties, which may be common for CYP with anxiety and depression,^12 13^ may require more intensive approaches to treat parental mental health. However, for some parents, a reduction in self-care and increase in self-blame may be usefully addressed by specific components.^52^ Due to this lack of underlying conceptualisation of parental distress, future research should clearly specify proposed causes of parental distress and elucidate what components of an intervention seek to address these issues.

Another potential major explanation for the results is due to the number of multifaceted interventions reported in the included studies. There were a total of nine intervention types measuring nearly 30 different outcomes. It is clear that within the evidence landscape, there are a range of interventions targeting different needs of parents/carers. Some interventions specifically addressed parents’ cognitive schemes relating to their life satisfaction, or offered a psychoeducational approach for applying problem solving to parents’ own difficulties.^38^ Other approaches had an emphasis on family relationships, specifically addressing how CYP anxiety and depression impacted this for parents.^45^ There appears to be a need for greater work to conceptualise and evidence the factors associated with distress in parents of CYP with anxiety and depression, to fine-tune interventions to directly target putative mechanisms.

A dominant approach to parent interventions involves parent training, which indirectly addresses parental well-being by improving CYP outcomes through improved parenting behaviours via skills teaching and increased parenting self-efficacy.^53 54^ Parent training has some short-term effects in a general population of parents,^54^ with their impact on parents of CYP with anxiety and depression yet to be fully investigated.

Another potential explanation for the findings is the limited number of studies per synthesis, compounded by different study designs and mixed quality of studies for each synthesis. However, sensitivity analyses (ie, isolating between-group and within-group designs) established that study design alone is unlikely to explain results, as summary effects remained similar for all outcomes. The varying study quality was most evident for the depression outcome, with quality evenly distributed among the three global ratings. To improve future studies, better participant representation to reduce selection bias with clinician/researcher-based reporting of outcomes is needed.

The imprecise measurement of outcomes poses a potential issue. There were various validated scales used to assess the same outcomes such as stress and burden, where three different measures were employed. The variation could potentially affect the validity in synthesising the results from different scales. However, for anxiety and depression, the use of only two different scales among the studies does not explain the results.

The included evidence had noticeable limitations. Study populations were predominantly mothers and studies were often limited by selection bias. Most studies lacked detailed reporting of parental characteristics such as parental ethnicity, which hindered potential post hoc analyses. Additionally, the absence of follow-up data prevented conclusions regarding long-term effects of interventions.

The overall review process was highly rigorous and reproducible with two reviewers independently performing all stages of screening and data extraction. The decision to restrict eligibility of studies to English is a limitation. The global representation of both published and grey literature included in this review supported by the absence of publication bias is a notable strength.

In conclusion, interventions aimed at directly improving parental well-being for parents of CYP with anxiety and/or depression are ineffective. The necessity of direct support remains unclear; however, this relies on a lack of conceptualisation of the mechanisms underpinning parents’ distress in relation to their CYP’s mental health.

## Supplementary material

10.1136/bmjment-2024-301218 online supplemental file 1

10.1136/bmjment-2024-301218 online supplemental file 2

10.1136/bmjment-2024-301218 online supplemental material 1

10.1136/bmjment-2024-301218 online supplemental material 2

10.1136/bmjment-2024-301218 online supplemental material 3

## Data Availability

All data relevant to the study are included in the article or uploaded as supplementary information.
