# Supplementary material for: Systematic review and meta-analysis of interventions to improve outcomes for parents or carers of children with anxiety and/or depression
Source: BMJ Ment Health. 2024 Sep 25;27(1):e301218. doi: 10.1136/bmjment-2024-301218 (PMC11425941; doi:10.1136/bmjment-2024-301218)
Supplement: online supplemental file 1 [file bmjment-27-1-s001.pdf]

# A systematic review and meta-analysis of interventions to improve outcomes for parents or carers of children with anxiety and/or depression


[*]Anthony Tsang,[†] Dania Dahmash,[‡] [§]Gretchen Bjornstad,[¶]

Nikki Rutter,[‖] Aleem Nisar,[**]Francesca Horne,[††]Faith Martin[‡‡]


25/06/2024

---

All analyses were conducted using *R* Version 4.3.1 released 16 June 2023 alongside RStudio version 2023.06.2+561 (Mountain Hydrangea) released 3 December 2022. The following packages were downloaded and installed:

install.packages("dmetar") Version 0.0.9000 still under development as of 2019

install.packages("meta") Version 6.5-0 published 7 June 2023

install.packages("esc") version 0.5.1 published 12 April 2019

install.packages ("MOTE") version 1.0.2 published 10 April 2019

library(dmetar)

library(meta)

library(esc)

library(MOTE)

**Data and analytical method used to synthesise anxiety outcomes**

The corresponding 95% confidence intervals of transformed effects were used to compute a standard error (SE) using the following formula: SE = (upper limit − lower limit) / 3.92. Pre-calculated effect size data were used because to include between- and within- group studies and raw data was not available for all studies (e.g., Salari et al. 2018).

The function *esc_mean_sd* from the R package *esc* was used to calculate Cohen's *d* and the function *d.dep.t.avg* from the R package *MOTE* was used to calculate Cohen's *d*_av for between-group studies and


[*]This R Markdown document was produced by Anthony Tsang

[†]Division of Psychology & Mental Health, School of Health Sciences, University of Manchester, Zochonis building, Brunswick street, M13 9PL Manchester

[‡]Centre for Intelligent Healthcare, Coventry University, Richard Crossman Building, Jordan Well, Coventry CV1 5RW, UK

[§]The Medical School, University of Exeter, South Cloisters, St Luke's Campus, Heavitree Road, Exeter, EX1 2LU, UK

[¶]NIHR Applied Research Collaboration South West Peninsula (PenARC), University of Exeter, Exeter, UK

[‖]Department of Sociology, Durham University, 32 Old Elvet, Durham, DH1 3HN, UK

[**]Independent researcher

[††]Department of Social Work, Care and Communities School of Social Sciences, Nottingham Trent University, 50 Shakespeare St, Nottingham NG1 4FQ

[‡‡]School of Psychology, Cardiff University, 70 Park Place, Cardiff CF10 3AT

within-group studies, respectively. Post-intervention effects were calculated for the following studies:

*Bertino et al (2013)*

```
esc_mean_sd(grp1m = 27.2, grp1sd = 27.9, grp1n =39,
            grp2m = 37.9, grp2sd =35.1, grp2n =9, es.type = "d")
```

```
##
## Effect Size Calculation for Meta Analysis
##
##      Conversion: mean and sd to effect size d
##     Effect Size:  -0.3654
##  Standard Error:   0.3717
##        Variance:   0.1381
##        Lower CI:  -1.0939
##        Upper CI:   0.3630
##          Weight:   7.2389
```

*Gleeson et al. (2017)*

```
d.dep.t.avg(m1=8.65, m2=9.28, sd1=10.34, sd2=10.24, n=25, a = 0.05)
```

```
## $d
## [1] -0.06122449
##
## $dlow
## [1] -0.4529626
##
## $dhigh
## [1] 0.3317798
##
## $M1
## [1] 8.65
##
## $sd1
## [1] 10.34
##
## $se1
## [1] 2.068
##
## $M1low
## [1] 4.381858
##
## $M1high
## [1] 12.91814
##
## $M2
## [1] 9.28
##
## $sd2
## [1] 10.24
##
```

```
## $se2
## [1] 2.048
##
## $M2low
## [1] 5.053136
##
## $M2high
## [1] 13.50686
##
## $n
## [1] 25
##
## $df
## [1] 24
##
## $estimate
## [1] "$d_{av}$ = -0.06, 95\\% CI [-0.45, 0.33]"
```

*Poole et al. (2018)* reported standard errors (SEs) were first converted to standard deviations.

```r
1.31*sqrt(31) #grpn1
```

```
## [1] 7.293771
```

```r
1.35*sqrt(33) #grpn2
```

```
## [1] 7.75516
```

```r
esc_mean_sd(grp1m = 4.81, grp1sd = 7.29 , grp1n =31 ,
            grp2m = 6.71, grp2sd =7.76 , grp2n= 33 , es.type = "d")
```

*Salari et al. (2018)* reported a $t$-value from an independent samples $t$-test and this was used to estimate Cohen's $d$.

```r
esc_t(t = -0.58, grp1n =15 , grp2n =19, es.type="d")
```

```
##
## Effect Size Calculation for Meta Analysis
##
##      Conversion: t-value to effect size d
##     Effect Size:  -0.2003
##  Standard Error:   0.3462
##        Variance:   0.1199
##        Lower CI:  -0.8790
##        Upper CI:   0.4783
##          Weight:   8.3411
```

*Waters et al. (2009)*

```r
esc_mean_sd(grp1m = 1.65, grp1sd = 2.17, grp1n =25,
            grp2m = 1.25, grp2sd =1.98, grp2n =24, es.type = "d")
```

The corresponding 95% confidence intervals of transformed effects were used to compute a standard error (SE) using the following formula: SE = (upper limit – lower limit) / 3.92.

*Bertino et al.(2013)*

```r
(0.36 - (-1.09)) / 3.92
```

```
## [1] 0.369898
```

*Gleeson et al. (2017)*

```r
(0.33 - (-0.45)) / 3.92
```

*Poole et al. (2018)*

```r
(0.24 - (-0.74)) / 3.92
```

*Salari et al. (2018)*

```r
(0.48 - (-0.88)) / 3.92
```

*Waters et al. (2009)*

```r
(0.75 - (-0.37)) / 3.92
```

The function *metagen* from the R package *meta* was used to pool pre-calculated effect size data

Table of the data was generated using the *kable* function in *knitr*.

```r
kable(Anxiety_post_treatment_d)
```

| Author | TE | seTE | Within- or between-groups | Intervention type | CYP diagnosis | Study quality |
|---|---|---|---|---|---|---|
| Bertino et al. (2013) | -0.37 | 0.37 | Between | Family-based | Mixed | Moderate |
| Gleeson et al. (2017) | -0.06 | 0.20 | Within | "Social therapy" | Mixed | Weak |
| Poole et al. (2018) | -0.25 | 0.25 | Between | Family-based | Depression | Strong |
| Salari et al. (2018) | -0.20 | 0.35 | Between | Group CBT | Anxiety | Weak |
| Waters et al. (2009) | 0.19 | 0.29 | Between | Group CBT | Anxiety | Strong |

The inverse variance method using a random effects model was used to pool effect sizes due to anticipated high level of between-study heterogeneity. The summary measure produced was Cohen's *d* with the restricted maximum likelihood estimator (REML) used to estimate between-study variance. Knapp-Hartung adjustment was applied to calculate the confidence interval around the summary effect.

```r
Anxiety_PT_d <- metagen(TE = TE,
                 seTE = seTE,
                 studlab = Author,
                 data = Anxiety_post_treatment_d,
                 sm = "SMD",
                 fixed = FALSE,
                 random = TRUE,
                 method.tau = "REML",
                 prediction = TRUE,
                 hakn = TRUE,
                 title =
              "Effectiveness of interventions on parents'/carers' anxiety at post-intervention")
summary(Anxiety_PT_d)
```

```
## Review:     Effectiveness of interventions on parents'/carers' anxiety at post-intervention
##
##                             SMD            95%-CI %W(random)
## Bertino et al. (2013) -0.3700 [-1.0952; 0.3552]       10.7
## Gleeson et al. (2017) -0.0600 [-0.4520; 0.3320]       36.6
## Poole et al. (2018)   -0.2500 [-0.7400; 0.2400]       23.4
## Salari et al. (2018)  -0.2000 [-0.8860; 0.4860]       11.9
## Waters et al. (2009)   0.1900 [-0.3784; 0.7584]       17.4
##
## Number of studies: k = 5
##
##                                 SMD            95%-CI     t p-value
## Random effects model (HK) -0.1108 [-0.3486; 0.1270] -1.29  0.2654
## Prediction interval                [-0.4957; 0.2741]
##
## Quantifying heterogeneity:
##  tau^2 = 0 [0.0000; 0.2835]; tau = 0 [0.0000; 0.5324]
##  I^2 = 0.0% [0.0%; 79.2%]; H = 1.00 [1.00; 2.19]
##
## Test of heterogeneity:
##     Q d.f. p-value
##  2.01    4  0.7346
##
## Details on meta-analytical method:
## - Inverse variance method
## - Restricted maximum-likelihood estimator for tau^2
## - Q-Profile method for confidence interval of tau^2 and tau
## - Hartung-Knapp adjustment for random effects model (df = 4)
## - Prediction interval based on t-distribution (df = 3)
```

A forest plot was generated using the *forest.meta* function from the *meta* package using the meta-analysis output *Anxiety_PT_d*.

```r
forest.meta(Anxiety_PT_d, leftcols=c("studlab", "Intervention type", "CYP diagnosis", "Study quality"),
            rightcols=c("effect", "ci", "seTE", "w.random"),
            print.tau2 = FALSE,
            text.random = "Random effects model - Anxiety",
            print.pval.Q = FALSE,
            prediction =  TRUE,
```

```
            digits.TE= 2,
            digits.se= 2,
            label.left = "Favours intervention",
            label.right = "Favours control",
            col.diamond="blue",
            col.square="green", col.square.lines="black")
```

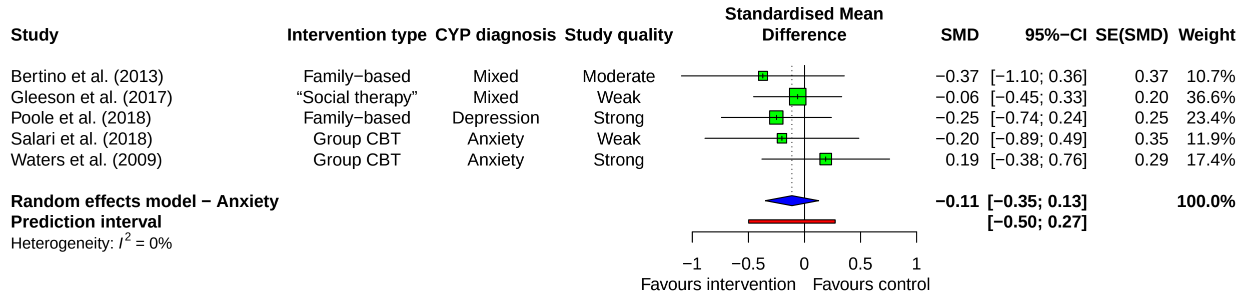


The function *find.outliers* part of the *dmetar* package was used to identify potential outliers (i.e., extremely small or large effects).

```
find.outliers(Anxiety_PT_d)
```

```
## No outliers detected (random-effects model).
```

Leave-one-out analysis was performed using the function *InfluenceAnalysis* part of the *dmetar* package. Influential cases have a large impact on the summary effect or heterogeneity.

```
InfluenceAnalysis(Anxiety_PT_d,random = TRUE)
```

```
## [===========================================================================] DONE
```

```
## Leave-One-Out Analysis (Sorted by I2)
##  -----------------------------------
##                              Effect   LLCI  ULCI I2
## Omitting Bertino et al. (2013) -0.080 -0.364 0.204  0
## Omitting Gleeson et al. (2017) -0.140 -0.525 0.245  0
## Omitting Poole et al. (2018)   -0.068 -0.390 0.253  0
## Omitting Salari et al. (2018)  -0.099 -0.428 0.230  0
## Omitting Waters et al. (2009)  -0.174 -0.379 0.031  0
##
##
## Influence Diagnostics
##  -------------------
##                              rstudent dffits cook.d cov.r QE.del   hat weight infl
## Omitting Bertino et al. (2013)   -0.741 -0.256  0.066 1.120  1.457 0.107 10.686
## Omitting Gleeson et al. (2017)    0.319  0.242  0.059 1.577  1.904 0.366 36.572
## Omitting Poole et al. (2018)     -0.636 -0.352  0.124 1.306  1.602 0.234 23.406
## Omitting Salari et al. (2018)    -0.271 -0.100  0.010 1.136  1.932 0.119 11.942
## Omitting Waters et al. (2009)     1.141  0.524  0.274 1.211  0.703 0.174 17.394
##
```

```
##
## Baujat Diagnostics (sorted by Heterogeneity Contribution)
##  -------------------------------------------------------
##                                  HetContrib InfluenceEffectSize
## Omitting Waters et al. (2009)       1.076               0.227
## Omitting Bertino et al. (2013)      0.491               0.059
## Omitting Poole et al. (2018)        0.310               0.095
## Omitting Gleeson et al. (2017)      0.065               0.037
## Omitting Salari et al. (2018)       0.065               0.009
```

Publication test was visually assessed through funnel plot asymmetry.

```
funnel(Anxiety_PT_d, xlab = "Funnel plot for anxiety at post-intervention", studlab = TRUE)
```

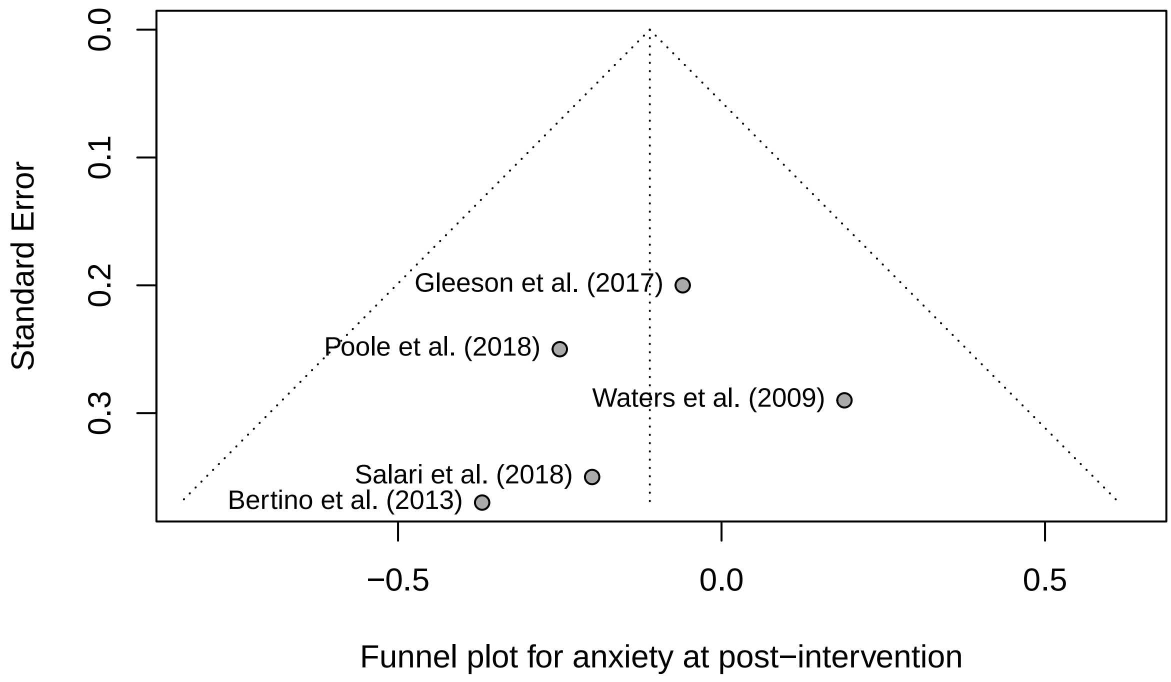


Asymmetry was quantified using Egger's test.

```
eggers.test(Anxiety_PT_d)
```

```
## Eggers' test of the intercept
## =============================
##
##  intercept       95% CI     t         p
##     -0.861 -3.88 - 2.15 -0.56 0.6146429
##
## Eggers' test does not indicate the presence of funnel plot asymmetry.
```

A sensitivity analysis was conducted that examined *between-group studies* only. This analysis used a corrected version of effects by applying Hedges' $g$ to the pre-calculated effect size data before pooling was conducted.

*Bertino et al.(2013)*

```
esc_mean_sd(grp1m = 27.2, grp1sd = 27.9, grp1n =39 ,
             grp2m = 37.9, grp2sd =35.1, grp2n =9, es.type = "g")
```

*Poole et al. (2018)*

```
esc_mean_sd(grp1m = 4.81, grp1sd = 7.29 , grp1n =31 ,
            grp2m = 6.71, grp2sd =7.76 , grp2n= 33 , es.type = "g")
```

*Salari et al. (2018)*

```
esc_t(t = -0.58, grp1n =15 , grp2n =19, es.type="g")
```

*Waters et al. (2009)*

```
esc_mean_sd(grp1m = 1.65, grp1sd = 2.17, grp1n =25,
            grp2m = 1.25, grp2sd =1.98, grp2n =24, es.type = "g")
```

The corresponding 95% confidence intervals of transformed effects were used to compute a SE.

*Bertino et al.(2013)*

```
(0.37 - (-1.09)) / 3.92
```

*Poole et al. (2018)*

```
(0.24 - (-0.74)) / 3.92
```

*Salari et al. (2018)*

```
(0.48 - (-0.87)) / 3.92
```

*Waters et al. (2009)*

```
(0.75 - (-0.37)) / 3.92
```

The data frame was tabulated.

| Author | TE | seTE | Within- or between-groups | Intervention type | CYP diagnosis | Study quality |
|---|---|---|---|---|---|---|
| Bertino et al. (2013) | -0.36 | 0.37 | Between | Family-based | Mixed | Moderate |
| Poole et al. (2018) | -0.25 | 0.25 | Between | Family-based | Depression | Strong |
| Salari et al. (2018) | -0.20 | 0.34 | Between | Group CBT | Anxiety | Weak |
| Waters et al. (2009) | 0.19 | 0.29 | Between | Group CBT | Anxiety | Strong |

A random effects model was ran estimating Hedges' $g$ by using REML as the estimator with Hartung-Knapp adjustment applied.

```
## Review:     Effectiveness of interventions on parents'/carers' anxiety at post-intervention
##
##                        Hedges' g            95%-CI %W(random)
## Bertino et al. (2013)   -0.3600 [-1.0852; 0.3652]       16.7
## Poole et al. (2018)     -0.2500 [-0.7400; 0.2400]       36.5
## Salari et al. (2018)    -0.2000 [-0.8664; 0.4664]       19.7
## Waters et al. (2009)     0.1900 [-0.3784; 0.7584]       27.1
##
## Number of studies: k = 4
##
##                            Hedges' g            95%-CI     t p-value
## Random effects model (HK)   -0.1391 [-0.5189; 0.2406] -1.17  0.3279
## Prediction interval                 [-0.7889; 0.5107]
##
## Quantifying heterogeneity:
##  tau^2 = 0 [0.0000; 0.7018]; tau = 0 [0.0000; 0.8377]
##  I^2 = 0.0% [0.0%; 84.7%]; H = 1.00 [1.00; 2.56]
##
## Test of heterogeneity:
##     Q d.f. p-value
##  1.87    3  0.5991
##
## Details on meta-analytical method:
## - Inverse variance method
## - Restricted maximum-likelihood estimator for tau^2
## - Q-Profile method for confidence interval of tau^2 and tau
## - Hartung-Knapp adjustment for random effects model (df = 3)
## - Prediction interval based on t-distribution (df = 2)
```

A forest plot was generated

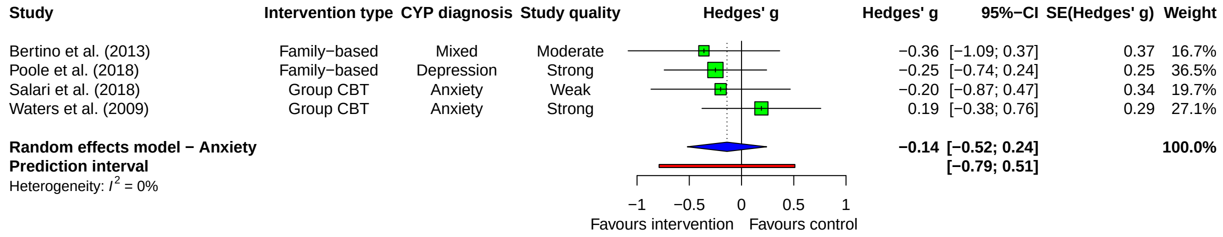


A test to detect outliers was performed

```
## No outliers detected (random-effects model).
```

Leave-one-out analysis was performed and identified *Waters et al. 2009* as an influential study on the summary effect

```
## [===========================================================================] DONE
```

```
## Leave-One-Out Analysis (Sorted by I2)
##  -----------------------------------
##                                Effect   LLCI   ULCI I2
```

```
## Omitting Bertino et al. (2013) -0.095 -0.700  0.510  0
## Omitting Poole et al. (2018)   -0.075 -0.796  0.645  0
## Omitting Salari et al. (2018)  -0.124 -0.819  0.570  0
## Omitting Waters et al. (2009)  -0.262 -0.437 -0.087  0
##
##
## Influence Diagnostics
##  -------------------
##                                rstudent dffits cook.d cov.r QE.del   hat weight infl
## Omitting Bertino et al. (2013)   -0.654 -0.292  0.085 1.200  1.446 0.167 16.660
## Omitting Poole et al. (2018)     -0.556 -0.422  0.178 1.575  1.563 0.365 36.492
## Omitting Salari et al. (2018)    -0.200 -0.099  0.010 1.246  1.833 0.197 19.729
## Omitting Waters et al. (2009)     1.329  0.811  0.658 1.372  0.106 0.271 27.119    *
##
##
## Baujat Diagnostics (sorted by Heterogeneity Contribution)
##  -------------------------------------------------------
##                                HetContrib InfluenceEffectSize
## Omitting Waters et al. (2009)       1.288               0.479
## Omitting Bertino et al. (2013)      0.356               0.071
## Omitting Poole et al. (2018)        0.197               0.113
## Omitting Salari et al. (2018)       0.032               0.008
```

Funnel plot was then generated to visually assess asymmetry for any potential publication bias

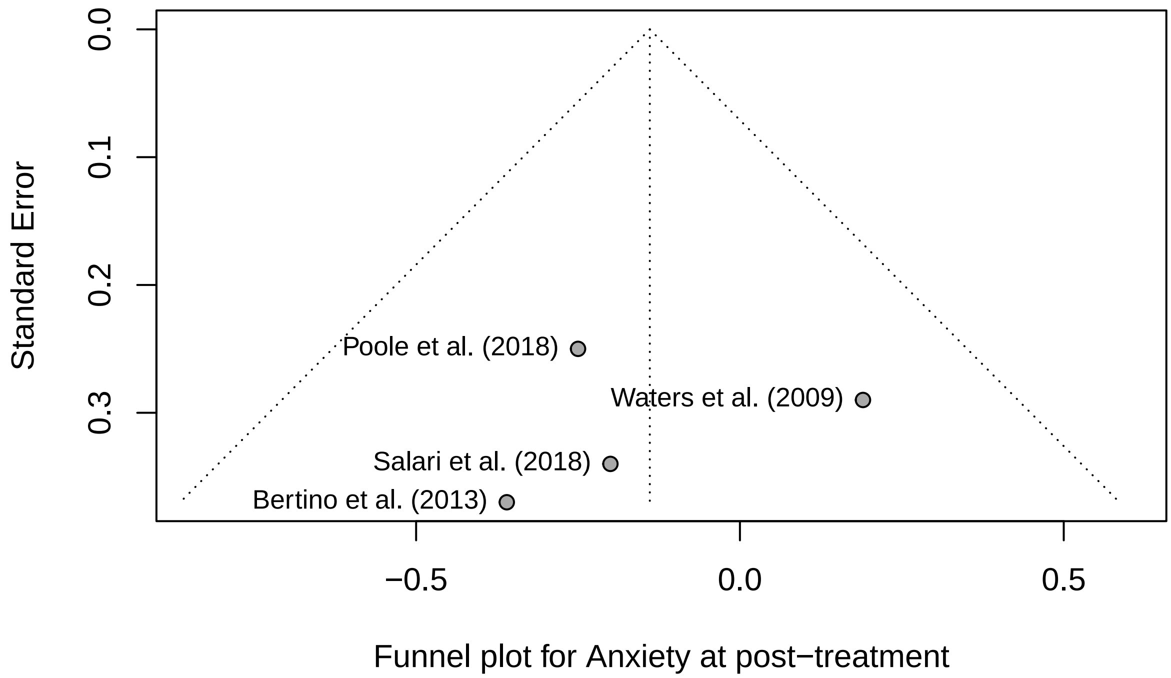


Egger's test was performed to statistically test for asymmetry

```
## Eggers' test of the intercept
```

```
## ============================
##
##  intercept       95% CI      t         p
##     -0.931 -7.09 - 5.23 -0.296 0.7951844
##
## Eggers' test does not indicate the presence of funnel plot asymmetry.
```

The analysis was re-ran to remove the influential case of *Waters et al. 2009* using the *update.meta* function part of the *meta* package

```
## Review:     Effectiveness of interventions on parents'/carers' anxiety at post-intervention
##
## Number of studies: k = 3
##
##                            Hedges' g             95%-CI     t p-value
## Random effects model (HK)   -0.2616 [-0.4366; -0.0866] -6.43  0.0233
## Prediction interval                 [-2.5093;  1.9861]
##
## Quantifying heterogeneity:
##  tau^2 = 0 [0.0000; 0.1378]; tau = 0 [0.0000; 0.3712]
##  I^2 = 0.0% [0.0%; 89.6%]; H = 1.00 [1.00; 3.10]
##
## Test of heterogeneity:
##     Q d.f. p-value
##  0.11    2  0.9485
##
## Details on meta-analytical method:
## - Inverse variance method
## - Restricted maximum-likelihood estimator for tau^2
## - Q-Profile method for confidence interval of tau^2 and tau
## - Hartung-Knapp adjustment for random effects model (df = 2)
## - Prediction interval based on t-distribution (df = 1)
```

Follow-up effects on anxiety were calculated using raw data from studies.

Raw data from the studies tabulated

| Author | n.e | mean.e | sd.e | n.c | mean.c | sd.c | Follow-up period | Intervention type | CYP diagnosis | Study quality |
|---|---|---|---|---|---|---|---|---|---|---|
| Bertino et al. (2013) | 24 | 36.50 | 26.30 | 2 | 36.00 | 33.9 | 6-month | Family-based | Mixed | Moderate |
| O'Brien (2007) | 6 | 8.70 | 1.60 | 6 | 11.20 | 2.1 | 1-month | Group CBT | Anxiety | Strong |
| Poole et al. (2018) | 31 | 2.94 | 7.79 | 33 | 7.18 | 8.1 | 3-month | Family-based | Depression | Strong |

The *metacont* function part of the *meta* package was used to meta-analyse raw data of eligible studies. An inverse variance method using a random effects model was ran with the REML as the estimator and Hartung-Knapp adjustments were applied. The bias-corrected Hedges' $g$ was the summary measure. Below is the meta-analysis for anxiety at follow-up.

```
## Review:     Effectiveness of interventions on parents'/carers' anxiety at follow-up
##
```

```
##                             SMD             95%-CI %W(random)
## Bertino et al. (2013)  0.0182 [-1.4243;  1.4607]        9.4
## O'Brien (2007)        -1.2357 [-2.5144;  0.0430]       12.0
## Poole et al. (2018)   -0.5268 [-1.0259; -0.0276]       78.6
##
## Number of studies: k = 3
## Number of observations: o = 102
##
##                            SMD            95%-CI     t p-value
## Random effects model -0.5604 [-1.4580; 0.3372] -2.69  0.1151
## Prediction interval           [-3.4296; 2.3089]
##
## Quantifying heterogeneity:
##  tau^2 < 0.0001 [0.0000; 15.1366]; tau = 0.0017 [0.0000; 3.8906]
##  I^2 = 0.0% [0.0%; 89.6%]; H = 1.00 [1.00; 3.10]
##
## Test of heterogeneity:
##     Q d.f. p-value
##  1.71    2  0.4259
##
## Details on meta-analytical method:
## - Inverse variance method
## - Restricted maximum-likelihood estimator for tau^2
## - Q-Profile method for confidence interval of tau^2 and tau
## - Hartung-Knapp adjustment for random effects model (df = 2)
## - Prediction interval based on t-distribution (df = 1)
## - Hedges' g (bias corrected standardised mean difference; using exact formulae)
```

A forest plot was generated for the results

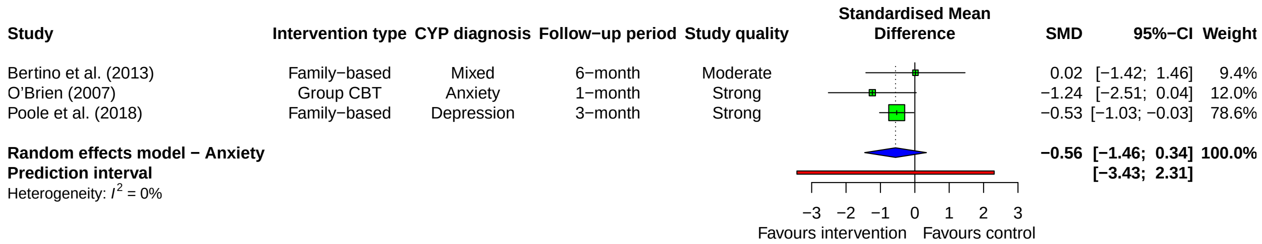


**Data and analytical method used to synthesise depression outcomes** Post-intervention effects were calculated for the following studies:

*Gerkensmeyer et al. (2013)* reported a *t*-value from an independent samples *t*-test and this was used to estimate Cohen's *d*.

```
esc_t(p = 0.0224, grp1n = 26, grp2n = 28, es.type = "d")
```

*Gleeson et al. (2017)*

```
d.dep.t.avg(m1=13.31, m2=12.31, sd1=16.15, sd2=12.43, n=25, a = 0.05)
```

*Poole et al. (2018)* reported SEs were first converted to standard deviations.

```r
2.05*sqrt(31) #grpn1
2.11*sqrt(33) #grpn2
```

```r
esc_mean_sd(grp1m = 10.87, grp1sd =11.41 , grp1n =31 ,
            grp2m = 10.95, grp2sd =12.12  , grp2n =33 , es.type = "d")
```

*Racey et al. (2018)*

```r
d.dep.t.avg(m1=6.7, m2=8.6, sd1=8.4, sd2=7.7, n=21, a = 0.05)
```

*Salari et al. (2018)* reported a *t*-value from an independent samples *t*-test and this was used to estimate Cohen's *d*.

```r
esc_t(t = 2.95, grp1n =15 , grp2n =19, es.type="d")
```

*Waters et al. (2009)*

```r
esc_mean_sd(grp1m = 1.65, grp1sd = 2.17, grp1n =25,
            grp2m = 1.25, grp2sd =1.98, grp2n =24, es.type = "d")
```

The corresponding 95% confidence intervals of transformed effects were used to compute SEs using the following formula: SE = (upper limit – lower limit) / 3.92.

*Gerkensmeyer et al. (2013)*

```r
(1.19 - (0.09)) / 3.92
```

*Gleeson et al. (2017)*

```r
(0.46 - (-0.32)) / 3.92
```

*Poole et al. (2018)*

```r
(0.48 - (-0.50)) / 3.92
```

*Racey et al. (2018)*

```r
(0.20 - (-0.67)) / 3.92
```

*Salari et al. (2018)*

```r
(1.71 - (0.28)) / 3.92
```

*Waters et al. (2009)*

```r
(0.75 - (-0.37)) / 3.92
```

Depression data at post-intervention.

| Author | TE | seTE | Within- or between-groups | Intervention type | CYP diagnosis | Study quality |
|---|---|---|---|---|---|---|
| Gerkensmeyer et al. (2013) | 0.64 | 0.28 | Between | Problem-solving | Mixed | Moderate |
| Gleeson et al. (2017) | 0.07 | 0.20 | Within | "Social therapy" | Mixed | Weak |
| Poole et al. (2018) | -0.01 | 0.25 | Between | Family-based | Depression | Strong |
| Racey et al. (2018) | -0.24 | 0.22 | Within | Mindfulness | Anxiety | Moderate |
| Salari et al. (2018) | 1.02 | 0.37 | Between | Group CBT | Anxiety | Weak |
| Waters et al. (2009) | 0.19 | 0.29 | Between | Group CBT | Anxiety | Strong |

Meta-analysis results for depression at post-intervention

```
## Review:     Effectiveness of interventions on parents'/carers' depression at post-intervention
##
##                                SMD            95%-CI %W(random)
## Gerkensmeyer et al. (2013)  0.6400 [ 0.0912; 1.1888]       15.9
## Gleeson et al. (2017)       0.0700 [-0.3220; 0.4620]       20.2
## Poole et al. (2018)        -0.0100 [-0.5000; 0.4800]       17.4
## Racey et al. (2018)        -0.2400 [-0.6712; 0.1912]       19.0
## Salari et al. (2018)        1.0200 [ 0.2948; 1.7452]       12.0
## Waters et al. (2009)        0.1900 [-0.3784; 0.7584]       15.4
##
## Number of studies: k = 6
##
##                              SMD            95%-CI    t p-value
## Random effects model (HK) 0.2204 [-0.2383; 0.6792] 1.24  0.2716
## Prediction interval              [-0.7889; 1.2297]
##
## Quantifying heterogeneity:
##  tau^2 = 0.1033 [0.0000; 1.2067]; tau = 0.3213 [0.0000; 1.0985]
##  I^2 = 59.4% [0.2%; 83.5%]; H = 1.57 [1.00; 2.46]
##
## Test of heterogeneity:
##      Q d.f. p-value
##  12.32    5  0.0307
##
## Details on meta-analytical method:
## - Inverse variance method
## - Restricted maximum-likelihood estimator for tau^2
## - Q-Profile method for confidence interval of tau^2 and tau
## - Hartung-Knapp adjustment for random effects model (df = 5)
## - Prediction interval based on t-distribution (df = 4)
```

A forest plot of the results.

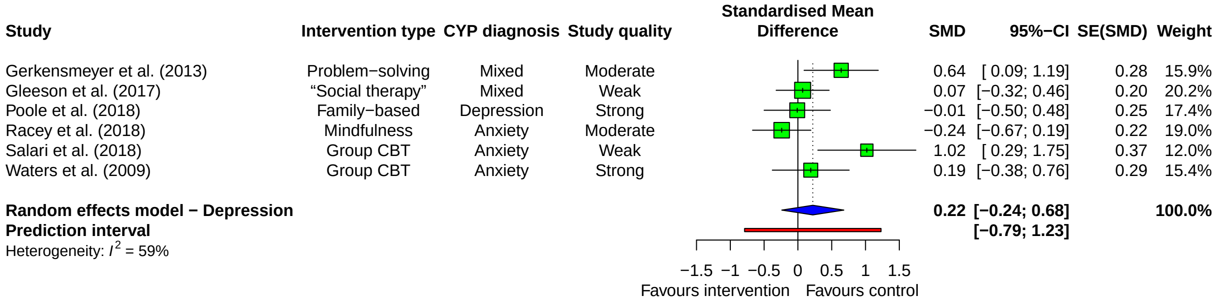


A test to identify outliers was conducted.

```
## No outliers detected (random-effects model).
```

Leave-one-out analysis was performed. *Salari et al. (2018)* was identified as an influential case because of its undue influence on both the summary effect and magnitude of heterogeneity.

```
## [===========================================================================] DONE
```

```
## Leave-One-Out Analysis (Sorted by I2)
##  -----------------------------------
##                                     Effect   LLCI  ULCI    I2
## Omitting Salari et al. (2018)        0.098 -0.292 0.487 0.374
## Omitting Racey et al. (2018)         0.320 -0.185 0.824 0.511
## Omitting Gerkensmeyer et al. (2013)  0.132 -0.395 0.660 0.548
## Omitting Poole et al. (2018)         0.281 -0.313 0.874 0.661
## Omitting Gleeson et al. (2017)       0.272 -0.342 0.885 0.669
## Omitting Waters et al. (2009)        0.240 -0.371 0.851 0.675
##
##
## Influence Diagnostics
##  -------------------
##                                     rstudent dffits cook.d cov.r QE.del   hat weight infl
## Omitting Gerkensmeyer et al. (2013)    1.180  0.560  0.269 1.007  8.846 0.159 15.904
## Omitting Gleeson et al. (2017)        -0.408 -0.256  0.090 1.629 12.074 0.202 20.167
## Omitting Poole et al. (2018)          -0.582 -0.317  0.125 1.497 11.795 0.174 17.429
## Omitting Racey et al. (2018)          -1.434 -0.649  0.341 1.027  8.185 0.190 19.050
## Omitting Salari et al. (2018)          2.122  0.857  0.521 0.644  6.391 0.120 12.030    *
## Omitting Waters et al. (2009)         -0.094 -0.102  0.013 1.541 12.304 0.154 15.420
##
##
## Baujat Diagnostics (sorted by Heterogeneity Contribution)
##  -------------------------------------------------------
##                                     HetContrib InfluenceEffectSize
## Omitting Salari et al. (2018)            5.466               0.463
## Omitting Racey et al. (2018)             3.223               0.913
## Omitting Gerkensmeyer et al. (2013)      3.001               0.473
## Omitting Poole et al. (2018)             0.435               0.090
## Omitting Gleeson et al. (2017)           0.180               0.066
## Omitting Waters et al. (2009)            0.015               0.002
```

Funnel plot was generated.

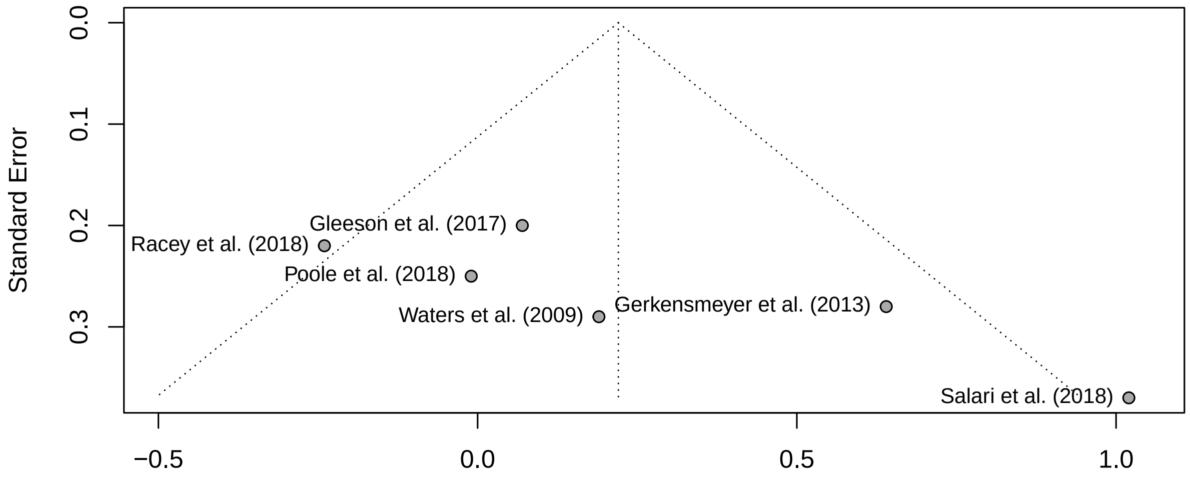


Funnel plot for depression at post−intervention

Egger's test was conducted.

```
## Eggers' test of the intercept
## =============================
##
##  intercept       95% CI     t          p
##      6.055 1.65 - 10.46 2.694 0.05445785
##
## Eggers' test does not indicate the presence of funnel plot asymmetry.
```

The meta-analysis was updated to remove the influential study.

```
## Review:     Effectiveness of interventions on parents'/carers' depression at post-intervention
##
## Number of studies: k = 5
##
##                               SMD            95%-CI    t p-value
## Random effects model (HK) 0.0978 [-0.2916; 0.4872] 0.70  0.5240
## Prediction interval              [-0.6272; 0.8228]
##
## Quantifying heterogeneity:
##  tau^2 = 0.0333 [0.0000; 0.8073]; tau = 0.1825 [0.0000; 0.8985]
##  I^2 = 37.4% [0.0%; 76.7%]; H = 1.26 [1.00; 2.07]
##
## Test of heterogeneity:
##     Q d.f. p-value
##  6.39    4  0.1718
##
## Details on meta-analytical method:
## - Inverse variance method
```

```
## - Restricted maximum-likelihood estimator for tau^2
## - Q-Profile method for confidence interval of tau^2 and tau
## - Hartung-Knapp adjustment for random effects model (df = 4)
## - Prediction interval based on t-distribution (df = 3)
```

A sensitivity analysis was conducted that examined *between-group studies* only. This analysis used a corrected version of effects by applying Hedges' $g$ to the pre-calculated effect size data before pooling was conducted.

*Gerkensmeyer et al. (2013)*

```
esc_t(p = 0.0224, grp1n = 26, grp2n = 28, es.type = "g")
```

*Poole et al. (2018)*

```
esc_mean_sd(grp1m = 10.87, grp1sd =11.41 , grp1n =31 ,
            grp2m = 10.95, grp2sd =12.12  , grp2n =33 , es.type = "g")
```

*Salari et al. (2018)*

```
esc_t(t = 2.95, grp1n =15 , grp2n =19, es.type="g")
```

*Waters et al. (2009)*

```
esc_mean_sd(grp1m = 1.65, grp1sd = 2.17, grp1n =25,
            grp2m = 1.25, grp2sd =1.98, grp2n =24, es.type = "g")
```

The corresponding 95% confidence intervals of transformed effects were used to compute SEs.

*Gerkensmeyer et al. (2013)*

```
(1.18 - (0.08)) / 3.92
```

*Poole et al. (2018)*

```
(0.48 - (-0.50)) / 3.92
```

*Salari et al. (2018)*

```
(1.74 - (0.30)) / 3.92
```

*Waters et al. (2009)*

```
(0.75 - (-0.37)) / 3.92
```

Table of depression data at post-intervention.

| Author | TE | seTE | Within- or between-groups | Intervention type | CYP diagnosis | Study quality |
|---|---|---|---|---|---|---|
| Gerkensmeyer et al. (2013) | 0.63 | 0.28 | Between | Problem-solving | Mixed | Moderate |
| Poole et al. (2018) | -0.01 | 0.25 | Between | Family-based | Depression | Strong |
| Salari et al. (2018) | 0.99 | 0.36 | Between | Group CBT | Anxiety | Weak |
| Waters et al. (2009) | 0.19 | 0.29 | Between | Group CBT | Anxiety | Strong |

Meta-analysis results for depression at post-treatment for between-group studies only.

```
## Review:     Effectiveness of interventions on parents'/carers' depression at post-intervention
##
##                              Hedges' g            95%-CI %W(random)
## Gerkensmeyer et al. (2013)    0.6300 [ 0.0812; 1.1788]       26.0
## Poole et al. (2018)          -0.0100 [-0.5000; 0.4800]       28.6
## Salari et al. (2018)          0.9900 [ 0.2844; 1.6956]       20.2
## Waters et al. (2009)          0.1900 [-0.3784; 0.7584]       25.2
##
## Number of studies: k = 4
##
##                             Hedges' g            95%-CI    t p-value
## Random effects model (HK)    0.4089 [-0.2863; 1.1040] 1.87  0.1580
## Prediction interval                 [-1.2307; 2.0484]
##
## Quantifying heterogeneity:
##  tau^2 = 0.0990 [0.0000; 2.6972]; tau = 0.3147 [0.0000; 1.6423]
##  I^2 = 54.0% [0.0%; 84.8%]; H = 1.47 [1.00; 2.56]
##
## Test of heterogeneity:
##     Q d.f. p-value
##  6.52    3  0.0887
##
## Details on meta-analytical method:
## - Inverse variance method
## - Restricted maximum-likelihood estimator for tau^2
## - Q-Profile method for confidence interval of tau^2 and tau
## - Hartung-Knapp adjustment for random effects model (df = 3)
## - Prediction interval based on t-distribution (df = 2)
```

A forest plot of the results.

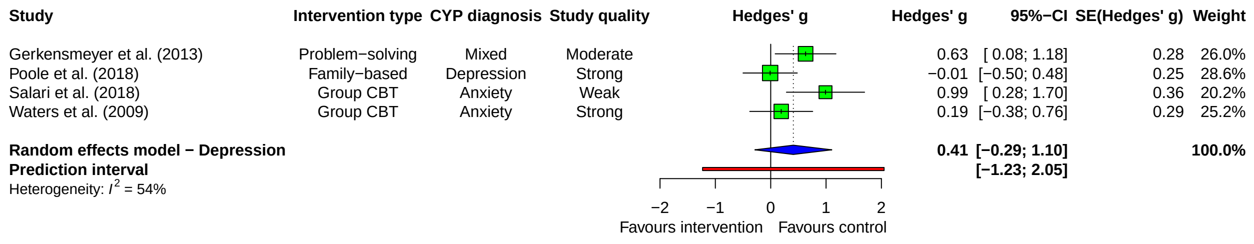


The detection for outliers was performed.

```
## No outliers detected (random-effects model).
```

Leave-one-out analyses identified *Poole et al. (2018)* and *Salari et al. (2018)* as influential cases that had a large impact on the level of heterogeneity.

```
## [===========================================================================] DONE
```

```
## Leave-One-Out Analysis (Sorted by I2)
##  -----------------------------------
##                                     Effect   LLCI  ULCI    I2
## Omitting Salari et al. (2018)        0.257 -0.563 1.077 0.326
## Omitting Poole et al. (2018)         0.569 -0.395 1.532 0.358
## Omitting Gerkensmeyer et al. (2013)  0.344 -0.926 1.615 0.625
## Omitting Waters et al. (2009)        0.497 -0.759 1.753 0.668
##
##
## Influence Diagnostics
##  -------------------
##                                     rstudent dffits cook.d cov.r QE.del   hat weight infl
## Omitting Gerkensmeyer et al. (2013)    0.507  0.261  0.090 1.779  5.330 0.260 26.019
## Omitting Poole et al. (2018)          -1.446 -0.892  0.553 1.038  3.116 0.286 28.580    *
## Omitting Salari et al. (2018)          1.623  0.828  0.500 0.805  2.966 0.202 20.192    *
## Omitting Waters et al. (2009)         -0.526 -0.348  0.169 1.861  6.027 0.252 25.209
##
##
## Baujat Diagnostics (sorted by Heterogeneity Contribution)
##  -------------------------------------------------------
##                                     HetContrib InfluenceEffectSize
## Omitting Salari et al. (2018)            2.990               0.568
## Omitting Poole et al. (2018)             2.280               1.127
## Omitting Gerkensmeyer et al. (2013)      0.879               0.315
## Omitting Waters et al. (2009)            0.375               0.122
```

Funnel plot was generated to allow for visual inspection for publication bias.

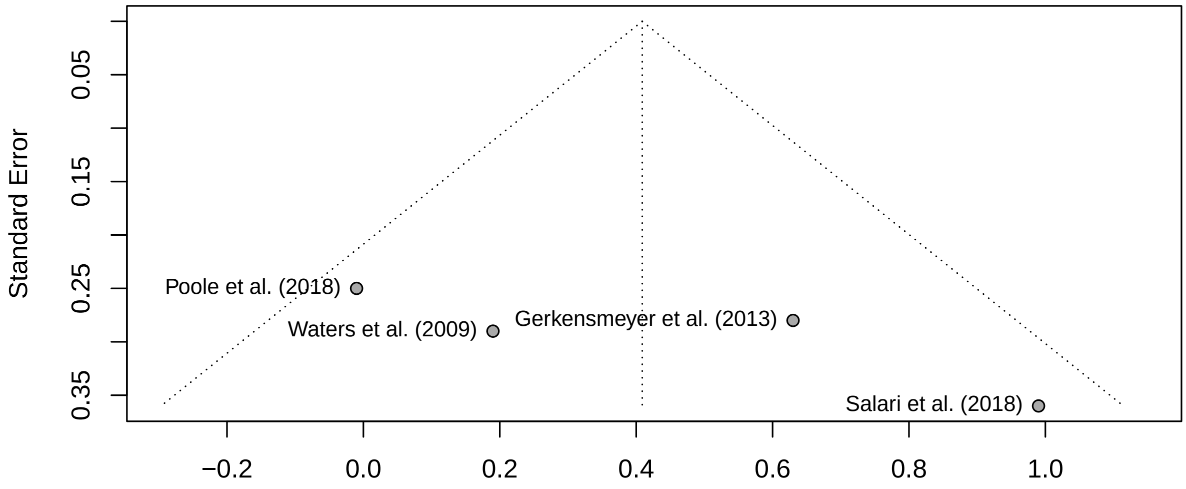


Funnel plot for depression at post−intervention

Egger's 'test was conducted to supplement visual inspection.

```
## Eggers' test of the intercept
## =============================
##
##  intercept       95% CI     t         p
##      8.557 1.05 - 16.06 2.235 0.1550066
##
## Eggers' test does not indicate the presence of funnel plot asymmetry.
```

The meta-analysis was re-ran without the two influential studies.

```
## Review:     Effectiveness of interventions on parents'/carers' depression at post-intervention
##
## Number of studies: k = 2
##
##                           Hedges' g            95%-CI    t p-value
## Random effects model (HK)    0.4165 [-2.3777; 3.2106] 1.89  0.3093
## Prediction interval
##
## Quantifying heterogeneity:
##  tau^2 = 0.0155; tau = 0.1247; I^2 = 16.1%; H = 1.09
##
## Test of heterogeneity:
##     Q d.f. p-value
##  1.19    1  0.2751
##
## Details on meta-analytical method:
## - Inverse variance method
## - Restricted maximum-likelihood estimator for tau^2
## - Hartung-Knapp adjustment for random effects model (df = 1)
```

Follow-up effects on depression were calculated using pre-calculated effect size data.

*Boxmeyer (2004)*

```
d.dep.t.avg(m1=19.2, m2=20.3, sd1=13.6, sd2=13.0, n=154, a = 0.05)
```

*Gerkensmeyer et al. (2013)*

```
esc_t(p = 0.0056, grp1n = 24, grp2n = 27, es.type = "d")
```

*Poole et al. (2018)* reported standard errors and was first converted to standard deviations.

```
2.16*sqrt(31)#grpn1
2.18*sqrt(33) #grpn2
```

```
esc_mean_sd(grp1m = 8.17, grp1sd = 12.03 , grp1n =31 ,
            grp2m = 13.37, grp2sd =12.52  , grp2n =33 , es.type = "d")
```

SEs were computed from 95% confidence intervals. *Boxmeyer (2004)*

```
(0.08 - (-0.24)) / 3.92
```

*Gerkensmeyer et al. (2013)*

```
(1.39 - (0.24)) / 3.92
```

*Poole et al. (2018)*

```
(0.07 - (-0.92)) / 3.92
```

Data frame for depression data at follow-up

| Author | TE | seTE | Follow-up period | Within- or between-groups | Intervention type | CYP diagnosis | Study quality |
|---|---|---|---|---|---|---|---|
| Boxmeyer (2004) | -0.08 | 0.08 | 6-month | Within | Mixed | Mixed | Moderate |
| Gerkensmeyer et al. (2013) | 0.80 | 0.29 | 3-month | Between | Problem-solving | Mixed | Moderate |
| Poole et al. (2018) | -0.42 | 0.25 | 3-month | Between | Family-based | Depression | Strong |

A random effects meta-analysis using the REML estimator with Hartung-Knapp modification applied.

```
## Review:     Effectiveness of interventions on parents'/carers' depression at follow-up
##
##                                 SMD             95%-CI %W(random)
## Boxmeyer (2004)             -0.0800 [-0.2368; 0.0768]       37.9
## Gerkensmeyer et al. (2013)   0.8000 [ 0.2316; 1.3684]       30.2
## Poole et al. (2018)         -0.4200 [-0.9100; 0.0700]       32.0
##
```

```
## Number of studies: k = 3
##
##                              SMD            95%-CI    t p-value
## Random effects model (HK) 0.0767 [-1.4321; 1.5855] 0.22  0.8472
## Prediction interval              [-8.0830; 8.2364]
##
## Quantifying heterogeneity:
##  tau^2 = 0.2974 [0.0343; 15.5834]; tau = 0.5453 [0.1853; 3.9476]
##  I^2 = 81.7% [43.2%; 94.1%]; H = 2.34 [1.33; 4.11]
##
## Test of heterogeneity:
##      Q d.f. p-value
##  10.92    2  0.0043
##
## Details on meta-analytical method:
## - Inverse variance method
## - Restricted maximum-likelihood estimator for tau^2
## - Q-Profile method for confidence interval of tau^2 and tau
## - Hartung-Knapp adjustment for random effects model (df = 2)
## - Prediction interval based on t-distribution (df = 1)
```

A forest plot for the meta-analytic results.

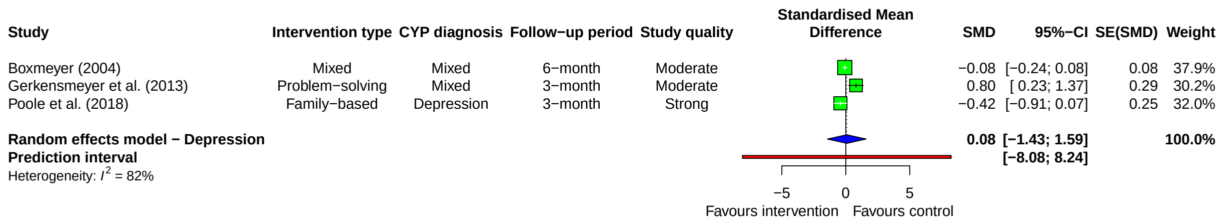


A sensitivity analysis was conducted that examined *between-group studies* only. This analysis used a corrected version of effects by applying Hedges' $g$ to the pre-calculated effect size data before pooling was conducted.

*Gerkensmeyer et al. (2013)*

```
esc_t(p = 0.0056, grp1n = 24, grp2n = 27, es.type = "g")
```

*Poole et al. (2018)*

```
esc_mean_sd(grp1m = 8.17, grp1sd = 12.03 , grp1n =31 ,
            grp2m = 13.37, grp2sd =12.52  , grp2n =33 , es.type = "g")
```

SEs were than estimated using 95% confidence intervals from the above effects

*Gerkensmeyer et al. (2013)*

```
(1.37 - (0.23)) / 3.92
```

*Poole et al. (2018)*

```r
(0.08 - (-0.91)) / 3.92
```

Data frame containing the pre-calculated effect size data of the studies

| Author | TE | seTE | Follow-up period | Within- or between-groups | Intervention type | CYP diagnosis | Study quality |
|---|---|---|---|---|---|---|---|
| Gerkensmeyer et al. (2013) | 0.80 | 0.29 | 3-month | Between | Problem-solving | Mixed | Moderate |
| Poole et al. (2018) | -0.42 | 0.25 | 3-month | Between | Family-based | Depression | Strong |

Meta-analysis results for depression at follow-up.

```
## Review:     Effectiveness of interventions on parents'/carers' depression at follow-up
##
##                              Hedges' g            95%-CI %W(random)
## Gerkensmeyer et al. (2013)    0.8000 [ 0.2316; 1.3684]       49.3
## Poole et al. (2018)          -0.4200 [-0.9100; 0.0700]       50.7
##
## Number of studies: k = 2
##
##                            Hedges' g            95%-CI    t p-value
## Random effects model (HK)    0.1811 [-7.5688; 7.9311] 0.30  0.8162
## Prediction interval
##
## Quantifying heterogeneity:
##  tau^2 = 0.6709; tau = 0.8191; I^2 = 90.2% [64.0%; 97.3%]; H = 3.19 [1.67; 6.09]
##
## Test of heterogeneity:
##      Q d.f. p-value
##  10.15    1  0.0014
##
## Details on meta-analytical method:
## - Inverse variance method
## - Restricted maximum-likelihood estimator for tau^2
## - Hartung-Knapp adjustment for random effects model (df = 1)
```

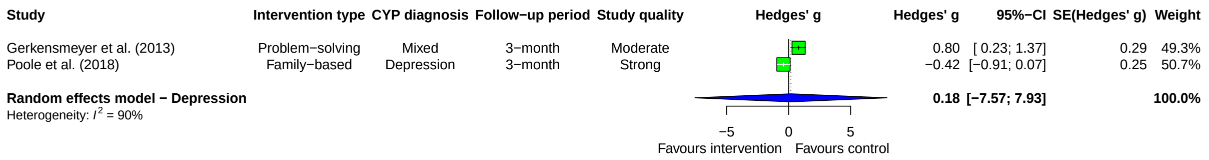


## Data and analytical method used to synthesise stress outcomes

Pre-calculated effect sizes were obtained for the following studies

*Gleeson et al. (2017)*

```
d.dep.t.avg(m1=19.07, m2=21.86, sd1=7.36, sd2=6.84, n=25, a = 0.05)
```

*Poole et al. (2018)* reported SEs were first converted to standard deviations.

```
1.68*sqrt(31) #grpn1
1.72*sqrt(33) #grpn2
```

```
esc_mean_sd(grp1m = 14.53 , grp1sd = 9.35 , grp1n =31 ,
            grp2m = 12.50 , grp2sd = 9.88, grp2n= 33 , es.type = "d")
```

*Reigstad et al. (2022)*

```
d.dep.t.avg(m1=237, m2=250.33, sd1=45.83, sd2=36.61, n=15, a = 0.05)
```

*Salari et al. (2016)*

```
esc_t(t = 1.10, grp1n =15, grp2n =19, es.type="d")
```

*Waters et al. (2009)*

```
esc_mean_sd(grp1m = 6.57, grp1sd = 5.75, grp1n =25,
            grp2m = 5.88, grp2sd =4.04, grp2n =24, es.type = "d")
```

SEs were than estimated from the corresponding 95% confidence intervals of the computed effects.

*Gleeson et al. (2017)*

```
(0.02 - (-0.80)) / 3.92
```

*Poole et al. (2018)*

```
(0.70 - (-0.28)) / 3.92
```

*Reigstad et al. (2022)*

```
(0.20 - (-0.84)) / 3.92
```

*Salari et al. (2016)*

```
(1.06 - (-0.30)) / 3.92
```

*Waters et al. (2009)*

```
(0.70 - (-0.42)) / 3.92
```

Data frame of studies investigating the effectiveness of interventions on stress.

| Author | TE | seTE | Within- or between-group | Intervention type | CYP diagnosis | Study quality |
|---|---|---|---|---|---|---|
| Gleeson et al. (2017) | -0.39 | 0.21 | Within | "Social therapy" | Mixed | Weak |
| Poole et al. (2018) | 0.21 | 0.25 | Between | Family-based | Depression | Strong |
| Reigtad et al. (2022) | -0.32 | 0.27 | Within | Parenting skills | Depression | Moderate |
| Salari et al. (2016) | 0.38 | 0.35 | Between | Group CBT | Anxiety | Weak |
| Waters et al. (2009) | 0.14 | 0.29 | Between | Group CBT | Anxiety | Strong |

Meta-analysis results for stress at post-intervention.

```
## Review:     Effectiveness of interventions on parents'/carers' stress at post-intervention
##
##                              SMD            95%-CI %W(random)
## Gleeson et al. (2017) -0.3900 [-0.8016; 0.0216]       26.1
## Poole et al. (2018)    0.2100 [-0.2800; 0.7000]       21.8
## Reigtad et al. (2022) -0.3200 [-0.8492; 0.2092]       19.9
## Salari et al. (2016)   0.3800 [-0.3060; 1.0660]       14.1
## Waters et al. (2009)   0.1400 [-0.4284; 0.7084]       18.2
##
## Number of studies: k = 5
##
##                                  SMD            95%-CI     t p-value
## Random effects model (HK) -0.0408 [-0.4616; 0.3800] -0.27  0.8010
## Prediction interval               [-0.8917; 0.8101]
##
## Quantifying heterogeneity:
##  tau^2 = 0.0476 [0.0000; 0.8776]; tau = 0.2181 [0.0000; 0.9368]
##  I^2 = 39.5% [0.0%; 77.6%]; H = 1.29 [1.00; 2.11]
##
## Test of heterogeneity:
##     Q d.f. p-value
##  6.61    4  0.1579
##
## Details on meta-analytical method:
## - Inverse variance method
## - Restricted maximum-likelihood estimator for tau^2
## - Q-Profile method for confidence interval of tau^2 and tau
## - Hartung-Knapp adjustment for random effects model (df = 4)
## - Prediction interval based on t-distribution (df = 3)
```

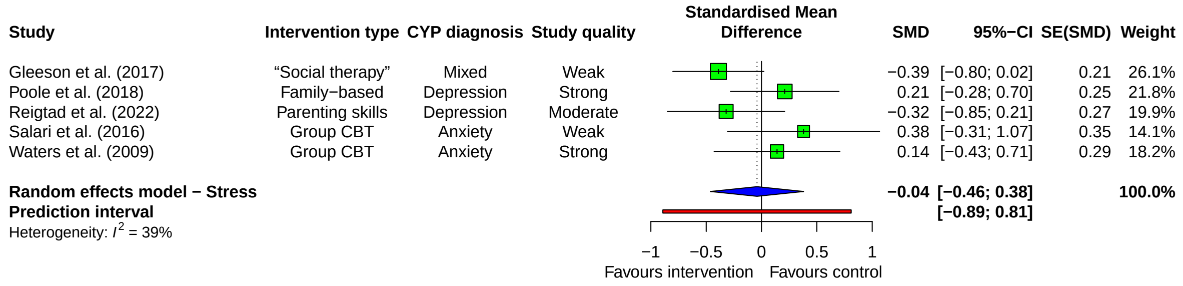

The detection of potential outliers was conducted.

```
## No outliers detected (random-effects model).
```

The test to identify influential cases was conducted. *Gleeson et al. (2017)* was detected as an influential case due to its influence on the summary effect and levels of heterogeneity.

```
## [===========================================================================] DONE
```

```
## Leave-One-Out Analysis (Sorted by I2)
##  ----------------------------------
##                                Effect   LLCI  ULCI    I2
## Omitting Gleeson et al. (2017)  0.077 -0.393 0.547 0.074
## Omitting Salari et al. (2016)  -0.112 -0.609 0.386 0.368
## Omitting Poole et al. (2018)   -0.110 -0.677 0.457 0.399
## Omitting Reigtad et al. (2022)  0.035 -0.516 0.585 0.460
## Omitting Waters et al. (2009)  -0.074 -0.668 0.519 0.498
##
##
## Influence Diagnostics
##  -------------------
##                                rstudent dffits cook.d cov.r QE.del   hat weight infl
## Omitting Gleeson et al. (2017)   -1.705 -1.003  0.578 0.933  3.241 0.261 26.118    *
## Omitting Poole et al. (2018)      0.851  0.447  0.201 1.287  4.996 0.218 21.751
## Omitting Reigtad et al. (2022)   -0.863 -0.461  0.238 1.407  5.558 0.199 19.873
## Omitting Salari et al. (2016)     1.137  0.470  0.210 1.070  4.744 0.141 14.076
## Omitting Waters et al. (2009)     0.496  0.201  0.047 1.441  5.981 0.182 18.182
##
##
## Baujat Diagnostics (sorted by Heterogeneity Contribution)
##  -------------------------------------------------------
##                                HetContrib InfluenceEffectSize
## Omitting Gleeson et al. (2017)      2.315               1.055
## Omitting Salari et al. (2016)       1.657               0.210
## Omitting Poole et al. (2018)        1.259               0.357
## Omitting Reigtad et al. (2022)      0.854               0.199
## Omitting Waters et al. (2009)       0.527               0.103
```

A funnel plot was generated.

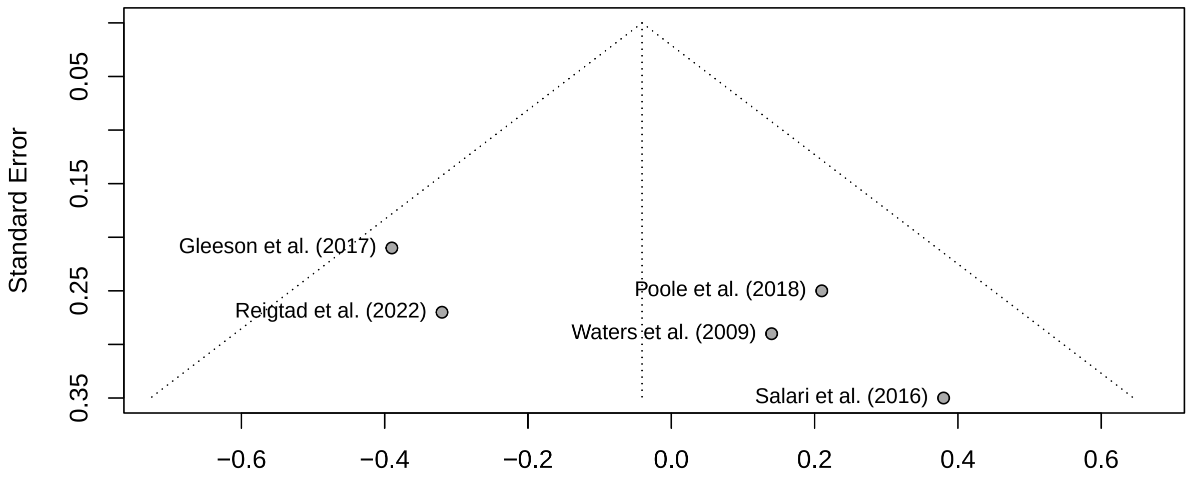


Funnel plot for stress at post−intervention

Egger's test was performed

```
## Eggers' test of the intercept
## =============================
##
##  intercept        95% CI     t        p
##      5.052 -0.37 - 10.47 1.827 0.165165
##
## Eggers' test does not indicate the presence of funnel plot asymmetry.
```

The analysis was updated to remove the influential case.

```
## Review:     Effectiveness of interventions on parents'/carers' stress at post-intervention
##
## Number of studies: k = 4
##
##                              SMD            95%-CI    t p-value
## Random effects model (HK) 0.0768 [-0.3930; 0.5466] 0.52  0.6390
## Prediction interval              [-0.6787; 0.8322]
##
## Quantifying heterogeneity:
##  tau^2 = 0.0085 [0.0000; 1.1577]; tau = 0.0922 [0.0000; 1.0760]
##  I^2 = 7.4% [0.0%; 85.8%]; H = 1.04 [1.00; 2.66]
##
## Test of heterogeneity:
##     Q d.f. p-value
##  3.24    3  0.3559
##
## Details on meta-analytical method:
## - Inverse variance method
## - Restricted maximum-likelihood estimator for tau^2
```

```
## - Q-Profile method for confidence interval of tau^2 and tau
## - Hartung-Knapp adjustment for random effects model (df = 3)
## - Prediction interval based on t-distribution (df = 2)
```

A sensitivity analysis was conducted that examined *between-group studies* only. This analysis used a corrected version of effects by applying Hedges' $g$ to the pre-calculated effect size data before pooling was conducted.

*Poole et al. (2018)*

```
esc_mean_sd(grp1m = 14.53 , grp1sd = 9.35 , grp1n =31 ,
            grp2m = 12.50 , grp2sd = 9.88, grp2n= 33 , es.type = "g")
```

*Salari et al. (2016)*

```
esc_t(t = 1.10, grp1n =15, grp2n =19, es.type="g")
```

*Waters et al. (2009)*

```
esc_mean_sd(grp1m = 6.57, grp1sd = 5.75, grp1n =25,
            grp2m = 5.88, grp2sd =4.04, grp2n =24, es.type = "g")
```

SEs were then estimated using the corresponding 95% confidence intervals of the computed effects above.

*Poole et al. (2018)*

```
(0.70 - (-0.28)) / 3.92
```

*Salari et al. (2016)*

```
(1.05 - (-0.30)) / 3.92
```

*Waters et al. (2009)*

```
(0.70 - (-0.42)) / 3.92
```

| Author | TE | seTE | Within- or between-groups | Intervention type | CYP diagnosis | Study quality |
|---|---|---|---|---|---|---|
| Poole et al. (2018) | 0.21 | 0.25 | Between | Family-based | Depression | Strong |
| Salari et al. (2016) | 0.37 | 0.34 | Between | Group CBT | Anxiety | Weak |
| Waters et al. (2009) | 0.14 | 0.29 | Between | Group CBT | Anxiety | Strong |

Meta-analysis results for stress at post-intervention for between-group studies only.

```
## Review:     Effectiveness of interventions on parents'/carers' stress at post-intervention
##
##                          SMD            95%-CI %W(random)
## Poole et al. (2018)  0.2100 [-0.2800; 0.7000]       43.8
## Salari et al. (2016) 0.3700 [-0.2964; 1.0364]       23.7
## Waters et al. (2009) 0.1400 [-0.4284; 0.7084]       32.5
```

```
##
## Number of studies: k = 3
##
##                               SMD            95%-CI    t p-value
## Random effects model (HK) 0.2251 [-0.0371; 0.4873] 3.69  0.0661
## Prediction interval              [-1.8769; 2.3271]
##
## Quantifying heterogeneity:
##  tau^2 = 0 [0.0000; 0.4465]; tau = 0 [0.0000; 0.6682]
##  I^2 = 0.0% [0.0%; 89.6%]; H = 1.00 [1.00; 3.10]
##
## Test of heterogeneity:
##     Q d.f. p-value
##  0.27    2  0.8731
##
## Details on meta-analytical method:
## - Inverse variance method
## - Restricted maximum-likelihood estimator for tau^2
## - Q-Profile method for confidence interval of tau^2 and tau
## - Hartung-Knapp adjustment for random effects model (df = 2)
## - Prediction interval based on t-distribution (df = 1)
```

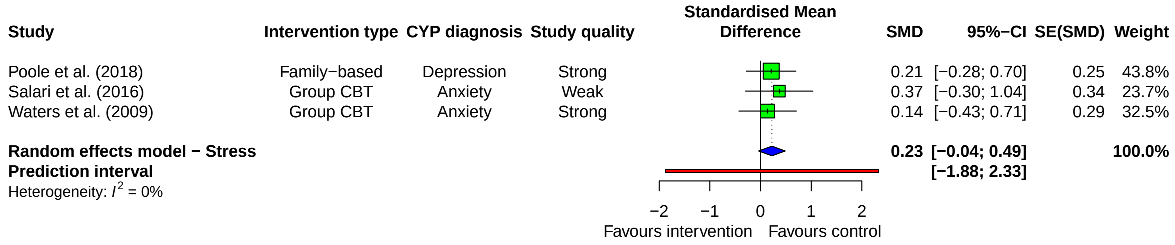


*Poole et al. (2018)* was the only study that reported an effect for stress at follow-up (3-month)

SEs were converted into standard deviations

```r
1.79*sqrt(31) #grpn1
1.79*sqrt(33) #grpn2
```

```r
esc_mean_sd(grp1m =10.04 , grp1sd = 9.97, grp1n =31 ,
            grp2m =14.86 , grp2sd =10.28 , grp2n= 33 , es.type = "d")
```

```
##
## Effect Size Calculation for Meta Analysis
##
##      Conversion: mean and sd to effect size d
##     Effect Size:  -0.4758
##  Standard Error:   0.2536
##        Variance:   0.0643
##        Lower CI:  -0.9729
##        Upper CI:   0.0214
##          Weight:  15.5450
```

**Data and analytical method used to synthesise burden outcomes**

Pre-calculated effect sizes were obtained for the following studies

*Boxmeyer (2004)*

```
d.dep.t.avg(m1=2.5, m2=2.9, sd1=0.8, sd2=0.8, n=155, a = 0.05)
```

*Gerkensmeyer et al. (2013)*

```
esc_t(p = 0.0022, grp1n = 26, grp2n = 28, es.type = "d")
```

*Khor et al. (2021)*

```
d.dep.t.avg(m1=42.55, m2=46.15, sd1=12.30, sd2=13.29, n=64, a = 0.05)
```

SEs were estimated using the corresponding 95% confidence intervals of the calculated effects above

*Boxmeyer (2004)*

```
(-0.33 - (-0.67)) / 3.92
```

*Gerkensmeyer et al. (2013)*

```
(1.57 - (0.42)) / 3.92
```

*Khor et al. (2021)*

```
(-0.03 - (-0.53)) / 3.92
```

Data frame of pre-calculated effect size data.

| Author | TE | seTE | Within- or between-groups | Intervention type | CYP diagnosis | Study quality |
|---|---|---|---|---|---|---|
| Boxmeyer (2004) | -0.50 | 0.09 | Within | Mixed | Mixed | Moderate |
| Gerkensmeyer et al. (2013) | 1.00 | 0.29 | Between | Problem-solving | Mixed | Moderate |
| Khor et al. (2021) | -0.28 | 0.13 | Within | Parent training | Anxiety and/or depression | Moderate |

Meta-analysis results for burden at post-intervention.

```
## Review:     Effectiveness of interventions on parents'/carers' burden at post-intervention
##
##                                  SMD             95%-CI %W(random)
## Boxmeyer (2004)             -0.5000 [-0.6764; -0.3236]       34.8
## Gerkensmeyer et al. (2013)   1.0000 [ 0.4316;  1.5684]       30.8
## Khor et al. (2021)          -0.2800 [-0.5348; -0.0252]       34.3
##
## Number of studies: k = 3
##
```

```
##                              SMD              95%-CI    t p-value
## Random effects model (HK) 0.0379 [ -1.9358;  2.0115] 0.08  0.9417
## Prediction interval              [-11.1589; 11.2346]
##
## Quantifying heterogeneity:
##  tau^2 = 0.5738 [0.1198; 25.8572]; tau = 0.7575 [0.3461; 5.0850]
##  I^2 = 91.9% [79.5%; 96.8%]; H = 3.52 [2.21; 5.60]
##
## Test of heterogeneity:
##      Q d.f.  p-value
##  24.72    2 < 0.0001
##
## Details on meta-analytical method:
## - Inverse variance method
## - Restricted maximum-likelihood estimator for tau^2
## - Q-Profile method for confidence interval of tau^2 and tau
## - Hartung-Knapp adjustment for random effects model (df = 2)
## - Prediction interval based on t-distribution (df = 1)
```

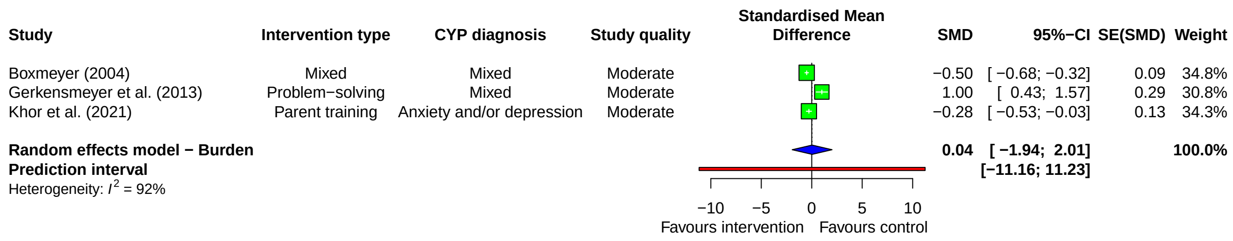


A sensitivity analysis examining *within-group* studies only was conducted.

*Boxmeyer (2004)*

```
d.dep.t.avg(m1=2.5, m2=2.9, sd1=0.8, sd2=0.8, n=155, a = 0.05)
```

*Khor et al. (2021)*

```
d.dep.t.avg(m1=42.55, m2=46.15, sd1=12.30, sd2=13.29, n=64, a = 0.05)
```

SEs were estimated using the corresponding 95% confidence intervals of the calculated effects above.

*Boxmeyer (2004)*

```
(-0.33 - (-0.67)) / 3.92
```

*Khor et al. (2021)*

```
(-0.03 - (-0.53)) / 3.92
```

Table of the pre-calculated effects.

| Author | TE | seTE | Within- or between-groups | Intervention type | CYP diagnosis | Study quality |
|---|---|---|---|---|---|---|
| Boxmeyer (2004) | -0.50 | 0.09 | Within | Mixed | Mixed | Moderate |
| Khor et al. (2021) | -0.28 | 0.13 | Within | Parent training | Anxiety and/or depression | Moderate |

Meta-analysis results for burden pertaining to within-group studies only.

```
## Review:     Effectiveness of interventions on parents'/carers' burden at post-intervention
##
##                          SMD             95%-CI %W(random)
## Boxmeyer (2004)    -0.5000 [-0.6764; -0.3236]       59.1
## Khor et al. (2021) -0.2800 [-0.5348; -0.0252]       40.9
##
## Number of studies: k = 2
##
##                                 SMD            95%-CI     t p-value
## Random effects model (HK) -0.4100 [-1.7844; 0.9644] -3.79  0.1642
## Prediction interval
##
## Quantifying heterogeneity:
##  tau^2 = 0.0117; tau = 0.1082; I^2 = 48.3%; H = 1.39
##
## Test of heterogeneity:
##     Q d.f. p-value
##  1.94    1  0.1641
##
## Details on meta-analytical method:
## - Inverse variance method
## - Restricted maximum-likelihood estimator for tau^2
## - Hartung-Knapp adjustment for random effects model (df = 1)
```

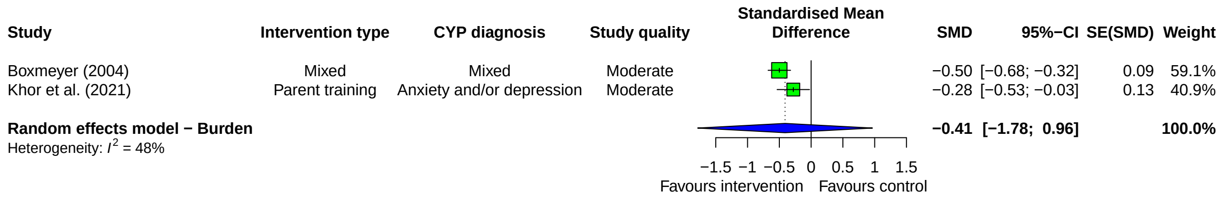


## Data and analytical method used to synthesise self-efficacy outcomes

Pre-calculated effect size data were obtained for the following studies

*Khor et al. (2021)*

```
d.dep.t.avg(m1=27.27, m2=22.52, sd1=3.49, sd2=4.80, n=64, a = 0.05)
```

*Reigstad et al. (2022)*

```r
d.dep.t.avg(m1=53.47, m2=48.40, sd1=6.64, sd2=6.30, n=15, a = 0.05)
```

*Waters et al. (2009)*

```r
esc_mean_sd(grp1m = 27.54, grp1sd = 8.57, grp1n =25,
            grp2m = 27.2, grp2sd =7.58, grp2n =24, es.type = "d")
```

SEs were estimated using the corresponding 95% confidence intervals of the calculated effects above.

*Khor et al. (2021)*

```r
(1.46 - (0.83)) / 3.92
```

*Reigstad et al. (2022)*

```r
(1.36 - (0.19)) / 3.92
```

*Waters et al. (2009)*

```r
(0.60 - (-0.52)) / 3.92
```

Table of pre-calculated effect size data.

| Author | TE | seTE | Within- or between-groups | Intervention type | CYP diagnosis | Study quality |
|---|---|---|---|---|---|---|
| Khor et al. (2021) | 1.15 | 0.16 | Within | Parent training | Anxiety and/or depression | Moderate |
| Reigstad et al. (2022) | 0.78 | 0.30 | Within | Attachment parenting | Depression | Moderate |
| Waters et al. (2009) | 0.04 | 0.29 | Between | CBT | Anxiety | Strong |

Meta-analysis results for self-efficacy at post-intervention.

```
## Review:     Effectiveness of interventions on parents'/carers' self-efficacy at post-intervention
##
##                          SMD            95%-CI %W(random)
## Khor et al. (2021)     1.1500 [ 0.8364; 1.4636]       37.7
## Reigstad et al. (2022) 0.7800 [ 0.1920; 1.3680]       30.9
## Waters et al. (2009)   0.0400 [-0.5284; 0.6084]       31.4
##
## Number of studies: k = 3
##
##                             SMD            95%-CI    t p-value
## Random effects model (HK) 0.6873 [-0.7234; 2.0979] 2.10  0.1710
## Prediction interval              [-7.0797; 8.4543]
##
## Quantifying heterogeneity:
##  tau^2 = 0.2643 [0.0287; 12.5550]; tau = 0.5141 [0.1694; 3.5433]
##  I^2 = 82.4% [45.8%; 94.3%]; H = 2.38 [1.36; 4.17]
```

```
##
## Test of heterogeneity:
##      Q d.f. p-value
##  11.34    2  0.0034
##
## Details on meta-analytical method:
## - Inverse variance method
## - Restricted maximum-likelihood estimator for tau^2
## - Q-Profile method for confidence interval of tau^2 and tau
## - Hartung-Knapp adjustment for random effects model (df = 2)
## - Prediction interval based on t-distribution (df = 1)
```

Forest plot for the outcome self-efficacy.

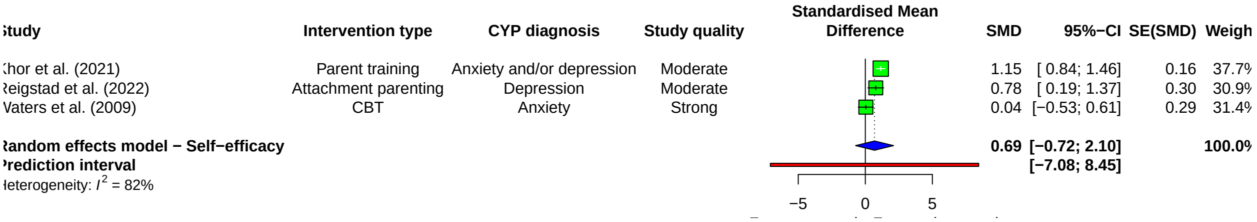


A sensitivity analysis examining *within-group* studies only was conducted.

*Khor et al. (2021)*

```
d.dep.t.avg(m1=27.27, m2=22.52, sd1=3.49, sd2=4.80, n=64, a = 0.05)
```

*Reigstad et al. (2022)*

```
d.dep.t.avg(m1=53.47, m2=48.40, sd1=6.64, sd2=6.30, n=15, a = 0.05)
```

```
## $d
## [1] 0.7836167
##
## $dlow
## [1] 0.1910806
##
## $dhigh
## [1] 1.355149
##
## $M1
## [1] 53.47
##
## $sd1
## [1] 6.64
##
## $se1
## [1] 1.714441
##
## $M1low
## [1] 49.79289
##
## $M1high
```

```
## [1] 57.14711
##
## $M2
## [1] 48.4
##
## $sd2
## [1] 6.3
##
## $se2
## [1] 1.626653
##
## $M2low
## [1] 44.91118
##
## $M2high
## [1] 51.88882
##
## $n
## [1] 15
##
## $df
## [1] 14
##
## $estimate
## [1] "$d_{av}$ = 0.78, 95\\% CI [0.19, 1.36]"
```

SEs were estimated using the corresponding 95% confidence intervals of the calculated effects above.

*Khor et al. (2021)*

```
(1.46 - (0.83)) / 3.92
```

*Reigstad et al. (2022)*

```
(1.36 - (0.19)) / 3.92
```

Table of pre-calculated effect size data.

| Author | TE | seTE | Within- or between-groups | Intervention type | CYP diagnosis | Study quality |
|---|---|---|---|---|---|---|
| Khor et al. (2021) | 1.15 | 0.16 | Within | Parent training | Anxiety and/or depression | Moderate |
| Reigstad et al. (2022) | 0.78 | 0.30 | Within | Attachment parenting | Depression | Moderate |

Meta-analysis results for self-efficacy at post-intervention.

```
## Review:     Effectiveness of interventions on parents'/carers' self-efficacy at post-intervention
##
##                            SMD           95%-CI %W(random)
## Khor et al. (2021)     1.1500 [0.8364; 1.4636]       73.5
## Reigstad et al. (2022) 0.7800 [0.1920; 1.3680]       26.5
```

```
##
## Number of studies: k = 2
##
##                                SMD             95%-CI    t p-value
## Random effects model (HK) 1.0520 [-1.0223; 3.1263] 6.44  0.0980
## Prediction interval
##
## Quantifying heterogeneity:
##  tau^2 = 0.0106; tau = 0.1032; I^2 = 15.6%; H = 1.09
##
## Test of heterogeneity:
##     Q d.f. p-value
##  1.18    1  0.2765
##
## Details on meta-analytical method:
## - Inverse variance method
## - Restricted maximum-likelihood estimator for tau^2
## - Hartung-Knapp adjustment for random effects model (df = 1)
```

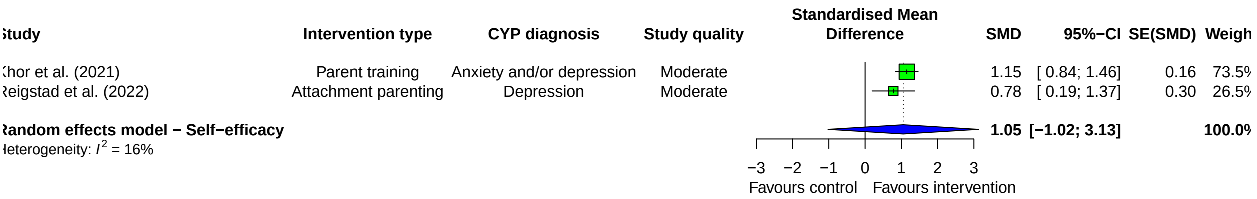


#### Data and analytical method used to synthesise quality of life outcomes
Pre-calculated effect size were obtained from the following studies

*Abedi and Vostanis (2010)*

```
esc_mean_sd(grp1m = 2.85, grp1sd = 1.18, grp1n =20 ,
            grp2m = 0.60, grp2sd =1.69, grp2n =20, es.type = "d")
```

*Gleeson et al. (2017)*

```
d.dep.t.avg(m1=126.48, m2=131.90, sd1=37.83, sd2=37.75, n=25, a = 0.05)
```

SEs were then calculated using the corresponding 95% confidence intervals from the calculated effects above

*Abedi and Vostanis (2010)*

```
(2.25 - (0.84)) / 3.92
```

*Gleeson et al. (2017)*

```
(0.25 - (-0.54)) / 3.92
```

Data frame of data of included studies investigating quality of life.

| Author | TE | seTE | Within- or between-groups | Intervention type | CYP diagnosis | Study quality |
|---|---|---|---|---|---|---|
| Abedi and Vostanis (2010) | 1.54 | 0.36 | Between | Family-based | OCD | Moderate |
| Gleeson et al. (2017) | -0.14 | 0.20 | Within | “Social therapy” | Mixed | Weak |

Meta-analysis results for quality of life.

```
## Review:     Effectiveness of interventions on parents’/carers’ quality of life at post-intervention
##
##                                 SMD            95%-CI %W(random)
## Abedi and Vostanis (2010)  1.5400 [ 0.8344; 2.2456]       48.4
## Gleeson et al. (2017)     -0.1400 [-0.5320; 0.2520]       51.6
##
## Number of studies: k = 2
##
##                                SMD             95%-CI    t p-value
## Random effects model (HK) 0.6733 [-9.9945; 11.3412] 0.80  0.5697
## Prediction interval
##
## Quantifying heterogeneity:
##  tau^2 = 1.3264; tau = 1.1517; I^2 = 94.0% [80.9%; 98.1%]; H = 4.08 [2.29; 7.27]
##
## Test of heterogeneity:
##      Q d.f.  p-value
##  16.64    1 < 0.0001
##
## Details on meta-analytical method:
## - Inverse variance method
## - Restricted maximum-likelihood estimator for tau^2
## - Hartung-Knapp adjustment for random effects model (df = 1)
```

Forest plot was generated.

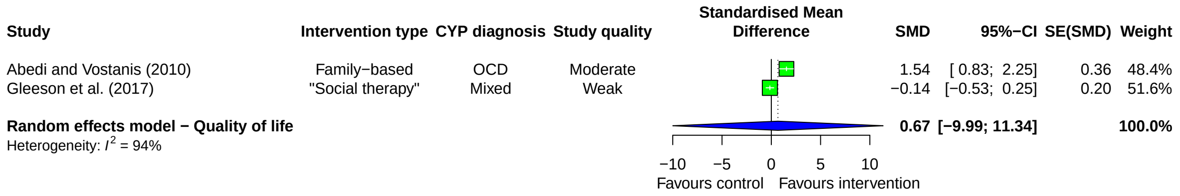


### Data and analytical method used to synthesise knowledge of mood disorders outcomes

Pre-calculated effect size were obtained from the following studies at post-intervention.

*Fristad et al (2003)*

```
esc_mean_sd(grp1m = 36.3, grp1sd = 4.1, grp1n =18,
            grp2m = 37.4, grp2sd =2.1, grp2n =13, es.type = "d")
```

*MacPherson et al. (2016)*

```
d.dep.t.avg(m1=36.11, m2=31.25, sd1=3.95, sd2=6.29, n=27, a = 0.05)
```

SEs were then calculated using the corresponding 95% confidence intervals from the calculated effects above

*Fristad et al (2003)*

```
(1.31 - (-0.11)) / 3.92
```

*MacPherson et al. (2016)*

```
(1.40 - (0.49)) / 3.92
```

Table depicting data pertaining to knowledge of mood disorders.

| Author | TE | seTE | Within- or between-groups | Intervention type | CYP diagnosis | Study quality |
|---|---|---|---|---|---|---|
| Fristad et al (2003) | 0.47 | 0.37 | Between | Psychoeducation | Biopolar depression | Moderate |
| MacPherson et al. (2016) | 0.95 | 0.23 | Within | Family therapy | Depression and bipolar | Moderate |

Meta-analysis results for knowledge of mood disorders at post-intervention.

```
## Review:     Effectiveness of interventions on parents'/carers' knowledge of
##                      mood disorders at post-intervention
##
##                               SMD            95%-CI %W(random)
## Fristad et al (2003)      0.4700 [-0.2552; 1.1952]       31.8
## MacPherson et al. (2016) 0.9500 [ 0.4992; 1.4008]       68.2
##
## Number of studies: k = 2
##
##                                SMD            95%-CI    t p-value
## Random effects model (HK) 0.7975 [-2.0421; 3.6371] 3.57  0.1739
## Prediction interval
##
## Quantifying heterogeneity:
##  tau^2 = 0.0203; tau = 0.1425; I^2 = 17.6%; H = 1.10
##
## Test of heterogeneity:
##     Q d.f. p-value
##  1.21    1  0.2706
##
## Details on meta-analytical method:
## - Inverse variance method
## - Restricted maximum-likelihood estimator for tau^2
## - Hartung-Knapp adjustment for random effects model (df = 1)
```

Forest plot depicting the meta-analysis results.

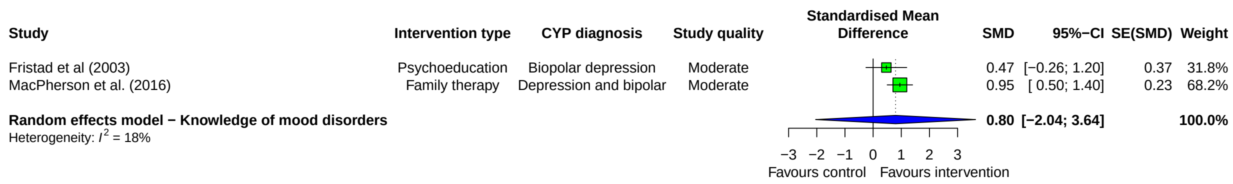


Knowledge of mood disorders at follow-up were calculated using pre-calculated effect size data.

*Fristad et al (2003)*

```
esc_mean_sd(grp1m = 36.9, grp1sd = 3.4, grp1n =16,
            grp2m = 33.8, grp2sd =6.5, grp2n =16, es.type = "d")
```

*MacPherson et al. (2016)* reported effects for two follow-up time points. The mean difference between six and twelve month follow-up were calculated using the below code

```
d.dep.t.avg(m1=36.25, m2=35.64, sd1=2.95, sd2=3.95, n=20, a = 0.05)
```

SEs were then calculated using the corresponding 95% confidence intervals from the calculated effects above

*Fristad et al (2003)*

```
(1.31 - (-0.11)) / 3.92
```

*MacPherson et al. (2016)*

```
(0.62 - (-0.27)) / 3.92
```

Data frame of follow-up data for knowledge of mood disorders.

| Author | TE | seTE | Within- or between-groups | Follow-up period | Intervention type | CYP diagnosis | Study quality |
|---|---|---|---|---|---|---|---|
| Fristad et al (2003) | 0.60 | 0.36 | Between | 4-month | Psychoeducation | Bipolar depression | Moderate |
| MacPherson et al. (2016) | 0.18 | 0.23 | Within | 6-month and 12-month | Family therapy | Depression and bipolar | Moderate |

Meta-analysis of knowledge of mood disorders at follow-up.

```
## Review:     Effectiveness of interventions on parents'/carers' knowledge of
##                      mood disorders at follow-up
##
##                                 SMD             95%-CI %W(random)
## Fristad et al (2003)      0.6000 [-0.1056; 1.3056]       29.0
## MacPherson et al. (2016) 0.1800 [-0.2708; 0.6308]       71.0
##
## Number of studies: k = 2
##
##                                  SMD             95%-CI    t p-value
```

```
## Random effects model (HK) 0.3017 [-2.1195; 2.7230] 1.58  0.3586
## Prediction interval
##
## Quantifying heterogeneity:
##  tau^2 = 0; tau = 0; I^2 = 0.0%; H = 1.00
##
## Test of heterogeneity:
##     Q d.f. p-value
##  0.97    1  0.3255
##
## Details on meta-analytical method:
## - Inverse variance method
## - Restricted maximum-likelihood estimator for tau^2
## - Hartung-Knapp adjustment for random effects model (df = 1)
```

Forest plot for knowledge of mood disorders at follow-up

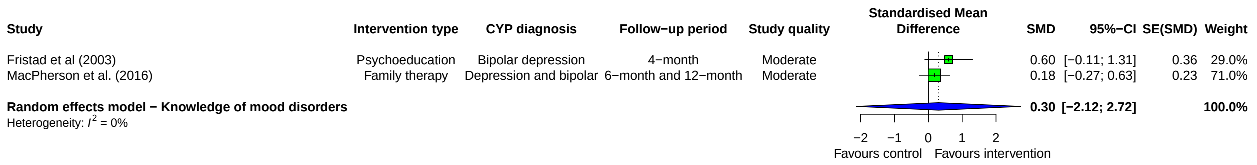


Below are parental outcomes that were only investigated by single studies. To standardise the reporting of effects for each outcome, between-group effects were either calculated using reported raw data or re-calculated using the *esc_mean_sd* or *esc_f*, or *esc_t* functions available in the *esc* package for consistency. For within-group studies, the *d.dep.t.avg* function was used from the *MOTE* package to calculate Cohen's *d*_av

Bertino et al. (2013)

Avoidance at post-intervention

```r
esc_mean_sd(grp1m = 35.6, grp1sd = 27.5, grp1n =39 ,
            grp2m = 39.2, grp2sd =28.2, grp2n =9, es.type = "g")
```

```
##
## Effect Size Calculation for Meta Analysis
##
##      Conversion: mean and sd to effect size Hedges' g
##     Effect Size:  -0.1282
##  Standard Error:   0.3700
##        Variance:   0.1369
##        Lower CI:  -0.8535
##        Upper CI:   0.5971
##          Weight:   7.3031
```

Avoidance at six-month follow-up

```r
esc_mean_sd(grp1m = 44.3, grp1sd = 26.8, grp1n =24 ,
            grp2m = 38.5, grp2sd =30.5, grp2n =2, es.type = "g")
```

```
##
## Effect Size Calculation for Meta Analysis
##
##      Conversion: mean and sd to effect size Hedges' g
##     Effect Size:   0.2083
##  Standard Error:   0.7366
##        Variance:   0.5426
##        Lower CI:  -1.2354
##        Upper CI:   1.6520
##          Weight:   1.8431
```

Bertino et al. (2013)

Compulsive traits at post-intervention

```r
esc_mean_sd(grp1m = 67, grp1sd = 17.5, grp1n =39 ,
            grp2m = 73.1, grp2sd =10.7, grp2n =9, es.type = "g")
```

```
##
## Effect Size Calculation for Meta Analysis
##
##      Conversion: mean and sd to effect size Hedges' g
##     Effect Size:  -0.3632
##  Standard Error:   0.3717
##        Variance:   0.1382
##        Lower CI:  -1.0918
##        Upper CI:   0.3653
##          Weight:   7.2373
```

Compulsive traits at six-month follow-up

```r
esc_mean_sd(grp1m = 60.2, grp1sd = 21.1, grp1n =24 ,
            grp2m = 69, grp2sd =15.6, grp2n =2, es.type = "g")
```

```
##
## Effect Size Calculation for Meta Analysis
##
##      Conversion: mean and sd to effect size Hedges' g
##     Effect Size:  -0.4078
##  Standard Error:   0.7383
##        Variance:   0.5451
##        Lower CI:  -1.8548
##        Upper CI:   1.0393
##          Weight:   1.8346
```

Bertino et al. (2013)

Avoidant attachment at post-intervention

```r
esc_mean_sd(grp1m = 21.7, grp1sd = 3.6, grp1n =38 ,
            grp2m = 23.2, grp2sd =2.2, grp2n =9, es.type = "g")
```

```
##
## Effect Size Calculation for Meta Analysis
##
##      Conversion: mean and sd to effect size Hedges' g
##     Effect Size:  -0.4346
##  Standard Error:   0.3735
##        Variance:   0.1395
##        Lower CI:  -1.1667
##        Upper CI:   0.2975
##          Weight:   7.1682
```

Bertino et al. (2013)

Anxious attachment at post-intervention

```
esc_mean_sd(grp1m = 9.9, grp1sd = 4.3, grp1n =39 ,
            grp2m = 12.2, grp2sd =5.2, grp2n =9, es.type = "g")
```

```
##
## Effect Size Calculation for Meta Analysis
##
##      Conversion: mean and sd to effect size Hedges' g
##     Effect Size:  -0.5062
##  Standard Error:   0.3735
##        Variance:   0.1395
##        Lower CI:  -1.2382
##        Upper CI:   0.2259
##          Weight:   7.1679
```

Fristad et al. (2003)

Positive expressed emotion at post-intervention

```
esc_mean_sd(grp1m = 119.3, grp1sd = 13.4, grp1n =18,
            grp2m = 111.5, grp2sd =13.8, grp2n =16, es.type = "g")
```

```
##
## Effect Size Calculation for Meta Analysis
##
##      Conversion: mean and sd to effect size Hedges' g
##     Effect Size:   0.5604
##  Standard Error:   0.3506
##        Variance:   0.1229
##        Lower CI:  -0.1267
##        Upper CI:   1.2475
##          Weight:   8.1366
```

Positive expressed emotion at 4-month follow-up

```
esc_mean_sd(grp1m = 121.7, grp1sd = 14.3, grp1n =16,
            grp2m = 104.4, grp2sd =19.1, grp2n =14, es.type = "g")
```

```
##
## Effect Size Calculation for Meta Analysis
##
##      Conversion: mean and sd to effect size Hedges' g
##     Effect Size:   1.0079
##  Standard Error:   0.3896
##        Variance:   0.1518
##        Lower CI:   0.2442
##        Upper CI:   1.7715
##          Weight:   6.5871
```

Fristad et al. (2003)

Negative expressed emotion at post-intervention

```
esc_mean_sd(grp1m = 66.9, grp1sd = 11.1, grp1n =18,
            grp2m = 72.9, grp2sd =12.1, grp2n =16, es.type = "g")
```

```
##
## Effect Size Calculation for Meta Analysis
##
##      Conversion: mean and sd to effect size Hedges' g
##     Effect Size:  -0.5059
##  Standard Error:   0.3493
##        Variance:   0.1220
##        Lower CI:  -1.1905
##        Upper CI:   0.1787
##          Weight:   8.1965
```

Negative expressed emotion at 4-month follow-up

```
esc_mean_sd(grp1m = 58.7, grp1sd = 16.5, grp1n =16,
            grp2m = 70.4, grp2sd =15.3, grp2n =14, es.type = "g")
```

```
##
## Effect Size Calculation for Meta Analysis
##
##      Conversion: mean and sd to effect size Hedges' g
##     Effect Size:  -0.7135
##  Standard Error:   0.3780
##        Variance:   0.1429
##        Lower CI:  -1.4544
##        Upper CI:   0.0274
##          Weight:   6.9983
```

Gerkensmeyer et al. (2013)

Perceived personal control at post-intervention

```
esc_f(f = 0.39, grp1n = 26, grp2n = 28, es.type="g")
```

```
##
```

```
## Effect Size Calculation for Meta Analysis
##
##      Conversion: F-value (one-way-Anova) to effect size Hedges' g
##     Effect Size:   0.1676
##  Standard Error:   0.2728
##        Variance:   0.0744
##        Lower CI:  -0.3671
##        Upper CI:   0.7024
##          Weight:  13.4330
```

Perceived personal control at 3-month follow-up

```r
esc_f(f=3.26, grp1n = 24, grp2n = 27, es.type="d")
```

```
##
## Effect Size Calculation for Meta Analysis
##
##      Conversion: F-value (one-way-Anova) to effect size d
##     Effect Size:   0.5065
##  Standard Error:   0.2850
##        Variance:   0.0812
##        Lower CI:  -0.0520
##        Upper CI:   1.0651
##          Weight:  12.3124
```

Gerkensmeyer et al. (2013)

Problem solving attitudes and skills at post-intervention

```r
esc_f(f = 1.61, grp1n = 26, grp2n = 28, es.type="d")
```

```
##
## Effect Size Calculation for Meta Analysis
##
##      Conversion: F-value (one-way-Anova) to effect size d
##     Effect Size:   0.3456
##  Standard Error:   0.2744
##        Variance:   0.0753
##        Lower CI:  -0.1922
##        Upper CI:   0.8833
##          Weight:  13.2835
```

Problem solving attitudes and skills at 3-month follow-up

```r
esc_f(f=0.03, grp1n = 24, grp2n = 27, es.type="d")
```

```
##
## Effect Size Calculation for Meta Analysis
##
##      Conversion: F-value (one-way-Anova) to effect size d
##     Effect Size:   0.0486
##  Standard Error:   0.2806
```

```
##        Variance:   0.0787
##        Lower CI:  -0.5013
##        Upper CI:   0.5985
##          Weight:  12.7021
```

Gleeson et al. (2017)

Perceived social support at post-intervention

```r
d.dep.t.avg(m1=4.00, m2=3.83, sd1=0.94, sd2=0.94, n=25, a = 0.05)
```

```
## $d
## [1] 0.1808511
##
## $dlow
## [1] -0.2162746
##
## $dhigh
## [1] 0.57429
##
## $M1
## [1] 4
##
## $sd1
## [1] 0.94
##
## $se1
## [1] 0.188
##
## $M1low
## [1] 3.611987
##
## $M1high
## [1] 4.388013
##
## $M2
## [1] 3.83
##
## $sd2
## [1] 0.94
##
## $se2
## [1] 0.188
##
## $M2low
## [1] 3.441987
##
## $M2high
## [1] 4.218013
##
## $n
## [1] 25
##
## $df
```

```
## [1] 24
##
## $estimate
## [1] "$d_{av}$ = 0.18, 95\\% CI [-0.22, 0.57]"
```

Khor et al. (2021)

Parent-adolescent attachment at post-intervention

```r
d.dep.t.avg(m1=63.97, m2=59.29, sd1=9.82, sd2=10.87, n=64, a = 0.05)
```

```
## $d
## [1] 0.4523925
##
## $dlow
## [1] 0.1933955
##
## $dhigh
## [1] 0.7081279
##
## $M1
## [1] 63.97
##
## $sd1
## [1] 9.82
##
## $se1
## [1] 1.2275
##
## $M1low
## [1] 61.51704
##
## $M1high
## [1] 66.42296
##
## $M2
## [1] 59.29
##
## $sd2
## [1] 10.87
##
## $se2
## [1] 1.35875
##
## $M2low
## [1] 56.57475
##
## $M2high
## [1] 62.00525
##
## $n
## [1] 64
##
## $df
```

```
## [1] 63
##
## $estimate
## [1] "$d_{av}$ = 0.45, 95\\% CI [0.19, 0.71]"
```

Khor et al. (2021)

Parental behaviours associated with reducing anxiety/depression in adolescents at post-intervention

```
d.dep.t.avg(m1=53.41, m2=47.77, sd1=7.40, sd2=7.51, n=64, a = 0.05)
```

```
## $d
## [1] 0.7565392
##
## $dlow
## [1] 0.4759754
##
## $dhigh
## [1] 1.032399
##
## $M1
## [1] 53.41
##
## $sd1
## [1] 7.4
##
## $se1
## [1] 0.925
##
## $M1low
## [1] 51.56153
##
## $M1high
## [1] 55.25847
##
## $M2
## [1] 47.77
##
## $sd2
## [1] 7.51
##
## $se2
## [1] 0.93875
##
## $M2low
## [1] 45.89406
##
## $M2high
## [1] 49.64594
##
## $n
## [1] 64
##
## $df
```

```
## [1] 63
##
## $estimate
## [1] "$d_{av}$ = 0.76, 95\\% CI [0.48, 1.03]"
```

MacPherson et al. (2016)

Treatment beliefs at post-intervention

```r
d.dep.t.avg(m1=4.00, m2=3.82, sd1=0.39, sd2=0.42, n=26, a = 0.05)
```

```
## $d
## [1] 0.4444444
##
## $dlow
## [1] 0.03697258
##
## $dhigh
## [1] 0.8438668
##
## $M1
## [1] 4
##
## $sd1
## [1] 0.39
##
## $se1
## [1] 0.07648529
##
## $M1low
## [1] 3.842476
##
## $M1high
## [1] 4.157524
##
## $M2
## [1] 3.82
##
## $sd2
## [1] 0.42
##
## $se2
## [1] 0.08236878
##
## $M2low
## [1] 3.650358
##
## $M2high
## [1] 3.989642
##
## $n
## [1] 26
##
## $df
```

```
## [1] 25
##
## $estimate
## [1] "$d_{av}$ = 0.44, 95\\% CI [0.04, 0.84]"
```

Treatment beliefs at six-month follow-up

```r
d.dep.t.avg(m1=3.90, m2=3.82, sd1=0.54, sd2=0.42, n=21, a = 0.05)
```

```
## $d
## [1] 0.1666667
##
## $dlow
## [1] -0.2661411
##
## $dhigh
## [1] 0.5953928
##
## $M1
## [1] 3.9
##
## $sd1
## [1] 0.54
##
## $se1
## [1] 0.1178377
##
## $M1low
## [1] 3.654195
##
## $M1high
## [1] 4.145805
##
## $M2
## [1] 3.82
##
## $sd2
## [1] 0.42
##
## $se2
## [1] 0.09165151
##
## $M2low
## [1] 3.628818
##
## $M2high
## [1] 4.011182
##
## $n
## [1] 21
##
## $df
## [1] 20
```

```
##
## $estimate
## [1] "$d_{av}$ = 0.17, 95\\% CI [-0.27, 0.60]"
```

Treatment beliefs at twelve-month follow-up

```r
d.dep.t.avg(m1=3.80, m2=3.82, sd1=0.59, sd2=0.42, n=20, a = 0.05)
```

```
## $d
## [1] -0.03960396
##
## $dlow
## [1] -0.4775266
##
## $dhigh
## [1] 0.3993528
##
## $M1
## [1] 3.8
##
## $sd1
## [1] 0.59
##
## $se1
## [1] 0.131928
##
## $M1low
## [1] 3.523872
##
## $M1high
## [1] 4.076128
##
## $M2
## [1] 3.82
##
## $sd2
## [1] 0.42
##
## $se2
## [1] 0.09391486
##
## $M2low
## [1] 3.623434
##
## $M2high
## [1] 4.016566
##
## $n
## [1] 20
##
## $df
## [1] 19
##
```

```
## $estimate
## [1] "$d_{av}$ = -0.04, 95\\% CI [-0.48, 0.40]"
```

Due to incompatible statistics, the following effects were not transformed from Pina (2005)

Anxiety at post-intervention (repeated measures ANOVA)

($F$(1,66), 16.89, $p$ <0.001; etq-squared = 0.20)

Anxiety at one year follow-up

($F$(1,45) = 5.73, $p < 0.05$; etq-squared = 0.1)

Racey et al. (2018)

Self-compassion at post-intervention

```
d.dep.t.avg(m1=3.4, m2=3.0, sd1=0.9, sd2=0.9, n=21, a = 0.05)
```

```
## $d
## [1] 0.4444444
##
## $dlow
## [1] -0.009631756
##
## $dhigh
## [1] 0.8884852
##
## $M1
## [1] 3.4
##
## $sd1
## [1] 0.9
##
## $se1
## [1] 0.1963961
##
## $M1low
## [1] 2.990325
##
## $M1high
## [1] 3.809675
##
## $M2
## [1] 3
##
## $sd2
## [1] 0.9
##
## $se2
## [1] 0.1963961
##
## $M2low
## [1] 2.590325
##
```

```
## $M2high
## [1] 3.409675
##
## $n
## [1] 21
##
## $df
## [1] 20
##
## $estimate
## [1] "$d_{av}$ = 0.44, 95\\% CI [-0.01, 0.89]"
```

Racey et al. (2018)

Mindfulness at post-intervention

```
d.dep.t.avg(m1=4.7, m2=3.9, sd1=0.8, sd2=1.2, n=8, a = 0.05)
```

```
## $d
## [1] 0.8
##
## $dlow
## [1] -0.02604859
##
## $dhigh
## [1] 1.585204
##
## $M1
## [1] 4.7
##
## $sd1
## [1] 0.8
##
## $se1
## [1] 0.2828427
##
## $M1low
## [1] 4.031183
##
## $M1high
## [1] 5.368817
##
## $M2
## [1] 3.9
##
## $sd2
## [1] 1.2
##
## $se2
## [1] 0.4242641
##
## $M2low
## [1] 2.896775
##
```

```
## $M2high
## [1] 4.903225
##
## $n
## [1] 8
##
## $df
## [1] 7
##
## $estimate
## [1] "$d_{av}$ = 0.80, 95\\% CI [-0.03, 1.59]"
```

Racey et al. (2018)

Decentring at post-intervention

```
d.dep.t.avg(m1=37.9, m2=31.3, sd1=6.5, sd2=8.5, n=17, a = 0.05)
```

```
## $d
## [1] 0.88
##
## $dlow
## [1] 0.3068579
##
## $dhigh
## [1] 1.433628
##
## $M1
## [1] 37.9
##
## $sd1
## [1] 6.5
##
## $se1
## [1] 1.576482
##
## $M1low
## [1] 34.55801
##
## $M1high
## [1] 41.24199
##
## $M2
## [1] 31.3
##
## $sd2
## [1] 8.5
##
## $se2
## [1] 2.061553
##
## $M2low
## [1] 26.9297
##
```

```
## $M2high
## [1] 35.6703
##
## $n
## [1] 17
##
## $df
## [1] 16
##
## $estimate
## [1] "$d_{av}$ = 0.88, 95\\% CI [0.31, 1.43]"
```

Racey et al. (2018)

Rumination at post-intervention

```r
d.dep.t.avg(m1=32.2, m2=39.3, sd1=9.8, sd2=15.5, n=20, a = 0.05)
```

```
## $d
## [1] -0.5612648
##
## $dlow
## [1] -1.027685
##
## $dhigh
## [1] -0.08219718
##
## $M1
## [1] 32.2
##
## $sd1
## [1] 9.8
##
## $se1
## [1] 2.191347
##
## $M1low
## [1] 27.61346
##
## $M1high
## [1] 36.78654
##
## $M2
## [1] 39.3
##
## $sd2
## [1] 15.5
##
## $se2
## [1] 3.465905
##
## $M2low
## [1] 32.04578
##
```

```
## $M2high
## [1] 46.55422
##
## $n
## [1] 20
##
## $df
## [1] 19
##
## $estimate
## [1] "$d_{av}$ = -0.56, 95\\% CI [-1.03, -0.08]"
```

Reigstad et al. (2022)

Stress at follow-up

```
d.dep.t.avg(m1=232.25, m2=250.33, sd1=45.93, sd2=36.61, n=15, a = 0.05)
```

```
## $d
## [1] -0.4380906
##
## $dlow
## [1] -0.9620286
##
## $dhigh
## [1] 0.09993468
##
## $M1
## [1] 232.25
##
## $sd1
## [1] 45.93
##
## $se1
## [1] 11.85908
##
## $M1low
## [1] 206.8148
##
## $M1high
## [1] 257.6852
##
## $M2
## [1] 250.33
##
## $sd2
## [1] 36.61
##
## $se2
## [1] 9.452661
##
## $M2low
## [1] 230.0561
##
```

```
## $M2high
## [1] 270.6039
##
## $n
## [1] 15
##
## $df
## [1] 14
##
## $estimate
## [1] "$d_{av}$ = -0.44, 95\\% CI [-0.96, 0.10]"
```

Reigstad et al. (2022)

Adolescent-parent relationship domain at post-intervention

```
d.dep.t.avg(m1=33.83, m2=37.75, sd1=6.87, sd2=5.55, n=15, a = 0.05)
```

```
## $d
## [1] -0.6312399
##
## $dlow
## [1] -1.178545
##
## $dhigh
## [1] -0.06544369
##
## $M1
## [1] 33.83
##
## $sd1
## [1] 6.87
##
## $se1
## [1] 1.773826
##
## $M1low
## [1] 30.02552
##
## $M1high
## [1] 37.63448
##
## $M2
## [1] 37.75
##
## $sd2
## [1] 5.55
##
## $se2
## [1] 1.433004
##
## $M2low
## [1] 34.67651
##
```

```
## $M2high
## [1] 40.82349
##
## $n
## [1] 15
##
## $df
## [1] 14
##
## $estimate
## [1] "$d_{av}$ = -0.63, 95\\% CI [-1.18, -0.07]"
```

Adolescent-parent relationship domain at follow-up

```r
d.dep.t.avg(m1=33.83, m2=37.75, sd1=9.74, sd2=5.55, n=15, a = 0.05)
```

```
## $d
## [1] -0.5127534
##
## $dlow
## [1] -1.044685
##
## $dhigh
## [1] 0.03513045
##
## $M1
## [1] 33.83
##
## $sd1
## [1] 9.74
##
## $se1
## [1] 2.514857
##
## $M1low
## [1] 28.43617
##
## $M1high
## [1] 39.22383
##
## $M2
## [1] 37.75
##
## $sd2
## [1] 5.55
##
## $se2
## [1] 1.433004
##
## $M2low
## [1] 34.67651
##
## $M2high
```

```
## [1] 40.82349
##
## $n
## [1] 15
##
## $df
## [1] 14
##
## $estimate
## [1] "$d_{av}$ = -0.51, 95\\% CI [-1.04, 0.04]"
```

Salari et al. (2018)

Global functioning at post-intervention

```r
esc_t(t = -2.11, grp1n =15, grp2n =19, es.type="g")
```

```
##
## Effect Size Calculation for Meta Analysis
##
##      Conversion: t-value to effect size Hedges' g
##     Effect Size:  -0.7116
##  Standard Error:   0.3565
##        Variance:   0.1271
##        Lower CI:  -1.4103
##        Upper CI:  -0.0128
##          Weight:   7.8673
```
