# Supplementary material for: Systematic review and meta-analysis of interventions to improve outcomes for parents or carers of children with anxiety and/or depression
Source: BMJ Ment Health. 2024 Sep 25;27(1):e301218. doi: 10.1136/bmjment-2024-301218 (PMC11425941; doi:10.1136/bmjment-2024-301218)
Supplement: online supplemental material 1 [file bmjment-27-1-s003.pdf]

All database and register searches were conducted on the 30th December 2023 by Faith Martin

1. **APA PsycInfo (EBSCO)**

Search	Terms
S1	TI (intervention or treatment or therapy or peer support or support group or psychotherapy or group support) OR AB (intervention or treatment or therapy or peer support or support group or psychotherapy or group support) OR (DE "Support Groups")) OR (DE "Peer Counseling")
S2	TI (Anxiety or depression or depressive or "obsessive compulsive disorder" or "OCD" or phobia or phobic or mood disorder or anxiety disorder or panic disorder or agoraphobia or internalising problem* or internalising disorder* or internalizing problem* or internalizing disorder*) OR AB (Anxiety or depression or depressive or "obsessive compulsive disorder" or "OCD" or phobia or phobic or mood disorder or anxiety disorder or panic disorder or agoraphobia or internalising problem* or internalising disorder* or internalizing problem* or internalizing disorder*) OR ((DE "Major Depression") OR (DE "Depression (Emotion)")) OR (DE "Anxiety Disorders")
S3	TI (children or adolescent* or adolescence or youth* or child or teenager* or kid* or teen* or young person or young people or boy* or girl* or juvenile*) OR AB (children or adolescent* or adolescence or youth* or child or teenager* or kid* or teen* or young person or young people or boy* or girl* or juvenile*)
S4	TI (parent or parents or parental or mother or father or guardian or carer* or paternal or maternal) OR AB (parent or parents or parental or mother or father or guardian or carer* or paternal or maternal) OR DE "Parents"
S5	TI (stress or anxious or anxiety or mental health or depression or depressive or burnout or "parenting satisfaction") OR AB (stress or anxious or anxiety or mental health or depression or depressive or burnout or "parenting satisfaction")
S6	1 and 2 and 3 and 4 and 5

2. **CINAHL Ultimate (EBSCO)**

Search	Terms
S1	TI (intervention or treatment or therapy or peer support or support group or psychotherapy or group support) OR AB (intervention or treatment or therapy or peer support or support group or psychotherapy or group support) OR (MH "Psychosocial Intervention") OR (MH "Support Groups") OR (MH "Peer Counseling") OR (MH "Peer Group")

S2	TI (Anxiety or depression or depressive or “obsessive compulsive disorder” or “OCD” or phobia or phobic or mood disorder or anxiety disorder or panic disorder or agoraphobia or internalising problem* or internalising disorder* or internalizing problem* or internalizing disorder*) OR AB (Anxiety or depression or depressive or “obsessive compulsive disorder” or “OCD” or phobia or phobic or mood disorder or anxiety disorder or panic disorder or agoraphobia or internalising problem* or internalising disorder* or internalizing problem* or internalizing disorder*) OR (MM "Depression+") OR (MM "Anxiety Disorders+")
S3	TI (children or adolescent* or adolescence or youth* or child or teenager* or kid* or teen* or young person or young people or boy* or girl* or juvenile*) OR AB (children or adolescent* or adolescence or youth* or child or teenager* or kid* or teen* or young person or young people or boy* or girl* or juvenile*)
S4	TI (parent or parents or parental or mother or father or caregiver or guardian or carer* or paternal or maternal) OR AB (parent or parents or parental or mother or father or caregiver or guardian or carer* or paternal or maternal) OR (MM "Parents+")
S5	TI (stress or anxious or anxiety or mental health or depression or depressive or burnout or "parenting satisfaction") OR AB (stress or anxious or anxiety or mental health or depression or depressive or burnout or "parenting satisfaction")
S6	(S4 N4 S1) AND (S5 N4 S4) AND (S3 N4 S2)

3. **Allied and Complementary Medicine (AMED) (EBSCO)**

Search	Terms
S1	TI (intervention or treatment or therapy or peer support or support group or psychotherapy or group support) OR AB (intervention or treatment or therapy or peer support or support group or psychotherapy or group support) OR (MH "Psychosocial Intervention") OR (MH "Psychotherapy") OR (MH "Self-Help Groups") OR (MH "Peer Group")
S2	TI (Anxiety or depression or depressive or “obsessive compulsive disorder” or “OCD” or phobia or phobic or mood disorder or anxiety disorder or panic disorder or agoraphobia or internalising problem* or internalising disorder* or internalizing problem* or internalizing disorder*) OR AB (Anxiety or depression or depressive or “obsessive compulsive disorder” or “OCD” or phobia or phobic or mood disorder or anxiety disorder or panic disorder or agoraphobia or internalising problem* or internalising disorder* or internalizing problem* or internalizing disorder*) OR (MH "Depressive Disorder") OR (MH "Depressive Disorder, Major") OR (MH "Depressive Disorder, Treatment-Resistant") OR (MH "Dysthymic Disorder") OR (MM "Anxiety Disorders+")

S3	TI (children or adolescent* or adolescence or youth* or child or teenager* or kid* or teen* or young person or young people or boy* or girl* or juvenile*) OR AB (children or adolescent* or adolescence or youth* or child or teenager* or pediatric* or paediatric* or kid* or teen* or young person or young people or boy* or girl* or juvenile*)
S4	TI (parent or parents or parental or mother or father or caregiver or guardian or carer* or paternal or maternal) OR AB (parent or parents or parental or mother or father or car*giver or guardian or carer* or paternal or maternal) OR (MM "Parents+")
S5	TI (stress or anxious or anxiety or mental health or depression or depressive or burnout or "parenting satisfaction") OR AB (stress or anxious or anxiety or mental health or depression or depressive or burnout or "parenting satisfaction")
S6	1 and 2 and 3 and 4 and 5

4. **MEDLINE (EBSCO)**

Search	Terms
S1	TI (intervention or treatment or therapy or peer support or support group or psychotherapy or group support) OR AB (intervention or treatment or therapy or peer support or support group or psychotherapy or group support) OR (MH "Psychosocial Intervention") OR (MH "Psychotherapy") OR (MH "Self-Help Groups") OR (MH "Peer Group")
S2	TI (Anxiety or depression or depressive or “obsessive compulsive disorder” or “OCD” or phobia or phobic or mood disorder or anxiety disorder or panic disorder or agoraphobia or internalising problem* or internalising disorder* or internalizing problem* or internalizing disorder*) OR AB (Anxiety or depression or depressive or “obsessive compulsive disorder” or “OCD” or phobia or phobic or mood disorder or anxiety disorder or panic disorder or agoraphobia or internalising problem* or internalising disorder* or internalizing problem* or internalizing disorder*) OR (MH "Depressive Disorder") OR (MH "Depressive Disorder, Major") OR (MH "Depressive Disorder, Treatment-Resistant") OR (MH "Dysthymic Disorder") OR (MM "Anxiety Disorders+")
S3	TI (children or adolescent* or adolescence or youth* or child or teenager* or kid* or teen* or young person or young people or boy* or girl* or juvenile*) OR AB (children or adolescent* or adolescence or youth* or child or teenager* or pediatric* or paediatric* or kid* or teen* or young person or young people or boy* or girl* or juvenile*)
S4	TI (parent or parents or parental or mother or father or caregiver or guardian or carer* or paternal or maternal) OR AB (parent or parents or parental or mother or father or car*giver or guardian or carer* or paternal or maternal) OR (MM "Parents+")
S5	TI (stress or anxious or anxiety or mental health or depression or depressive or burnout or "parenting satisfaction") OR AB (stress or

	anxious or anxiety or mental health or depression or depressive or burnout or "parenting satisfaction")
S6	(S4 N4 S1) AND (S6 N3 S4) AND (S3 N4 S2)

5. **EMBASE (Ovid)**

Search	Terms
1	(intervention or treatment or therapy or peer support or support group or psychotherapy or group support).ab. and (intervention or treatment or therapy or peer support or support group or psychotherapy or group support).ti.
2	(Anxiety or depression or depressive or obsessive compulsive disorder or OCD or phobia or phobic or mood disorder or anxiety disorder or panic disorder or agoraphobia or internalising problem* or internalising disorder* or internalizing problem* or internalizing disorder*).ab. and (Anxiety or depression or depressive or obsessive compulsive disorder or OCD or phobia or phobic or mood disorder or anxiety disorder or panic disorder or agoraphobia or internalising problem* or internalising disorder* or internalizing problem* or internalizing disorder*).ti.
3	(children or adolescent* or adolescence or youth* or child or teenager* or kid* or teen* or young person or young people or boy* or girl* or juvenile*).ab. and (children or adolescent* or adolescence or youth* or child or teenager* or kid* or teen* or young person or young people or boy* or girl* or juvenile*).ti.
4	(parent or parents or parental or mother or father or guardian or carer* or paternal or maternal).ab. and (parent or parents or parental or mother or father or guardian or carer* or paternal or maternal).ti.
5	(stress or anxious or anxiety or mental health or depression or depressive or burnout or parenting satisfaction).ab. and (stress or anxious or anxiety or mental health or depression or depressive or burnout or parenting satisfaction).ti.
6	(4 adj5 1) and (3 adj5 2) and 5

6. **Web of Science Core Collection (Science Citation Index Expanded [SCI-E], Social Sciences Citation Index [SSCI], Arts & Humanities Citation Index [A&HCI], Conference Proceedings Citation Index [CPCI-S], Conference Proceedings Citation Index- Social Sciences & Humanities [CPCI-SSH], Emerging Sources Citation Index [ESCI])**

Search	Terms
1	TI (intervention or treatment or therapy or peer support or support group or psychotherapy or group support) OR AB (intervention or treatment or therapy or peer support or support group or psychotherapy or group support)

2	TI (Anxiety or depression or depressive or “obsessive compulsive disorder” or “OCD” or phobia or phobic or mood disorder or anxiety disorder or panic disorder or agoraphobia or internalising problem* or internalising disorder* or internalizing problem* or internalizing disorder*) OR AB (Anxiety or depression or depressive or “obsessive compulsive disorder” or “OCD” or phobia or phobic or mood disorder or anxiety disorder or panic disorder or agoraphobia or internalising problem* or internalising disorder* or internalizing problem* or internalizing disorder*)
3	TI (children or adolescent* or adolescence or youth* or child or teenager* or kid* or teen* or young person or young people or boy* or girl* or juvenile*) OR AB (children or adolescent* or adolescence or youth* or child or teenager* or pediatric* or paediatric* or kid* or teen* or young person or young people or boy* or girl* or juvenile*)
4	TI (parent or parents or parental or mother or father or caregiver or guardian or carer* or paternal or maternal) OR AB (parent or parents or parental or mother or father or car*giver or guardian or carer* or paternal or maternal) OR (MM "Parents+")
5	TI (stress or anxious or anxiety or mental health or depression or depressive or burnout or "parenting satisfaction") OR AB (stress or anxious or anxiety or mental health or depression or depressive or burnout or "parenting satisfaction")
6	1 and 2 and 3 and 4 and 5

7. SCOPUS

Search	Terms
1	TITLE-ABS-KEY (intervention or treatment or therapy or peer support or support group or trial or psychotherapy or group support)
2	Anxiety or depression or depressive or “obsessive compulsive disorder” or “OCD” or phobia or phobic or mood disorder or anxiety disorder or panic disorder or agoraphobia or internalising problem* or internalising problem* or internalizing problem* or internalizing disorder*
3	children or adolescent* or adolescence or youth* or child or teenager* or pediatric* or paediatric* or kid* or teen* or young person or young people or boy* or girl* or juvenile*
4	parent or parents or parental or mother or father or care*giver or guardian or carer* or paternal or maternal
5	(s4 W/5 s1) AND (s3 W/5 s2)<br>Formatted as ((parent or parents or parental or mother or father or care*giver or guardian or carer* or paternal or maternal) W/5 (intervention or treatment or therapy or peer support or support group or trial or psychotherapy or group support)) AND ((children or adolescent* or adolescence or youth* or child or teenager* or pediatric* or paediatric* or kid* or teen* or young person or young people or boy* or girl* or juvenile*) W/5(Anxiety or depression or

	depressive or “obsessive compulsive disorder” or “OCD” or phobia or phobic or mood disorder or anxiety disorder or panic disorder or agoraphobia or internalising problem* or internalising problem* or internalizing problem* or internalizing disorder*))

8. **CENTRAL Cochrane Database**

(intervention or treatment or therapy or "peer support" or "support group" or psychotherapy or "group support"):ti,ab,kw AND (Anxiety or depression or depressive or “obsessive compulsive disorder” or “OCD” or phobia or phobic or mood disorder or anxiety disorder or panic disorder or agoraphobia or internalising problem* or internalising disorder* or internalizing problem* or internalizing disorder*):ti,ab,kw AND (children or adolescent* or adolescence or youth* or child or teenager* or kid* or teen* or "young person" or "young people" or boy* or girl* or juvenile*):ti,ab,kw AND (parent or parents or parental or mother or father or guardian or carer* or paternal or maternal):ti,ab,kw AND (stress or anxious or anxiety or mental health or depression or depressive or burnout or "parenting satisfaction"):ti,ab,kw

9. **WHO ICTRP**

The search was conducted using the Advanced Search interface

This search covers

- Australian New Zealand Clinical Trials Registry, last data file imported on **7 December 2023**
- Chinese Clinical Trial Registry, last data file imported on **7 December 2023**
- ClinicalTrials.gov, last data file imported on **6 December 2023**
- EU Clinical Trials Register (EU-CTR), last data file imported on **7 December 2023**
- ISRCTN, last data file imported on **30 November 2023**
- The Netherlands National Trial Register, last data file imported on **6 December 2023**
- Brazilian Clinical Trials Registry (ReBec), last data file imported on **6 December 2023**
- Clinical Trials Registry - India, last data file imported on **24 November 2023**
- Clinical Research Information Service - Republic of Korea, last data file imported on **2 December 2023**
- Cuban Public Registry of Clinical Trials, last data file imported on **29 November 2023**
- German Clinical Trials Register, last data file imported on **29 November 2023**
- Iranian Registry of Clinical Trials, last data file imported on **30 November 2023**
- Japan Primary Registries Network, last data file imported on **18 October 2023**
- Pan African Clinical Trial Registry, last data file imported on **29 November 2023**
- Sri Lanka Clinical Trials Registry, last data file imported on **29 November 2023**
- Thai Clinical Trials Registry (TCTR), last data file imported on **29 November 2023**
- Peruvian Clinical Trials Registry (REPEC), last data file imported on **8 November 2023**

- Lebanese Clinical Trials Registry (LBCTR), last data file imported on **29 November 2023**
