# Supplementary material for: Systematic review and meta-analysis of interventions to improve outcomes for parents or carers of children with anxiety and/or depression
Source: BMJ Ment Health. 2024 Sep 25;27(1):e301218. doi: 10.1136/bmjment-2024-301218 (PMC11425941; doi:10.1136/bmjment-2024-301218)
Supplement: online supplemental material 2 [file bmjment-27-1-s004.pdf]

Table 1 – Characteristics of eligible studies

Author, date, country	Study design	CYP diagnosis and age (years)	Participant characteristics and recruitment (where reported)	Parent outcome measures	Intervention name[a]	Intervention type
Abedi and Vostanis (2010),[1] Iran	RCT	OCD. 6 -18 (*m* = 11.75)	*N* = 40, 100% female, all mothers, recruited via schools and clinics	1. QoLI[2]	Quality of Life Therapy	Family-based
Bertino et al. (2013),[3] Australia	RCT	Anxiety, depression and/or substance misuse. 12-24 (*m* = 16.4, SD = 2.9)	*N* = 71, Age 33 to 64 years (*m* = 48, SD=6.8), 68% female – mothers, 32% fathers, recruited via clinical and community settings	1. MCMI-III-Anxiety[4]<br>2. MCMI-III-Avoidance[4]<br>3. MCMI-III-C[4]<br>4. RSQ-AvA[5]<br>5. RSQ-AnA[5]	BEST Plus	Family-based
Boxmeyer (2004),[6] USA	Quasi-experimental	ADHD, externalising disorders and anxiety. 11-18 (*m*= 13.5, *SD* = 2.0)	*N* = 157, Age 19 to 78 (*m* = 43.9, *SD* = 10.5), 93% female – mothers, 7% male – fathers, recruited via mental health clinics	1. CES-D[7]<br>2. CSQ[8]	Community based mental health treatment	Mixed
Fristad et al. (2003),[9] USA	RCT	Depressive and bipolar spectrum disorders. 8-11 (*m*= 10.1, *SD* = 1.2)	*N* = 47, 70% female - mothers, 21% fathers, 9% step-fathers, recruited via clinical and community based settings	1. UMDQ[10]<br>2. EEAC+[11]<br>3. EEAC-[11]	Multi-family psychoeducation	Psychoeducation
Gerkensmeyer et al. (2013),[12] USA	RCT (feasibility)	Psychological comorbidities including anxiety and depression. 11-16	*N* = 61, Age 32 to 69 (*m* = 42.7, SD = 9.2), 97% female, recruited via mental health centres.	1. BDI-II[13]<br>2. PES-B[14]<br>3. PMS[15]<br>4. SPSI-R:L[16]	Building Our Solutions and Connections	Problem-solving
Gleeson et al. (2017),[17] Australia	RCT (feasibility)	MDD, GAD, OCD, BP-II. *m* = 16.83, *SD* = 2.19	*N* = 29, Age *m* = 47.76, *SD* = 6.40, 86% female, parental role: Biological parent (97%) and guardian (*n* = 1), recruited via one-stop child mental health community centre	1. PSS[18]<br>2. DASS-A[19]<br>3. DASS-D[19]<br>4. SPWB[20]<br>5. MOSS-SSS[21]	Moderated Online Social Therapy	"Social therapy"
Khor et al. (2021),[22] Australia	RCT	Anxiety and/or depression. 12-18 (*m* = 15.02, *SD* 1.56)	*N* = 71, age *m* = 47.72, *SD* 5.14, 94% female, parental role: Biological parent (96%), Stepmother (3%),	1. PSES[23]<br>2. BAS[24]<br>3. K6[25]	Therapist-assisted Online Parenting Strategies Programme	Parent strategies

Author, date, country	Study design	CYP diagnosis and age (years)	Participant characteristics and recruitment (where reported)	Parent outcome measures	Intervention name[a]	Intervention type
			Grandmother (1%), 80% employed, recruited via community setting	4. P-A:A[26]<br>5. PRADAS[27]		
MacPherson et al. (2016),[28] USA	Open trial (pilot)	DD, bipolar disorder and cyclothymic disorder. 7-12 (*m* = 10.48, *SD* = 1.53)	*N* = 36, 95% female, parental role: Biological mother (75%), Adoptive mother (17%), Maternal biological father (6%), grandmother (3%), recruited via outpatient clinics	1. UMDQ[10]<br>2. TBQ-P[29]	Multi-Family Psychoeducational Psychotherapy	Group CBT
O'Brien et al. (2007),[30] Ireland	RCT	Dysthymia, social phobia, separation anxiety, panic with/without agoraphobia, elective mutism, OCD and GAD. 7-15 (*m* = 13.15, *SD* = 1.01)	*N*= 12, recruited via outpatient clinic	1. DASS-A[31]	Friends for Youth	Group CBT
Pina (2005),[32] USA	Randomised pre-post experimental	Anxiety and/or phobic disorder. 6-16 (*m*= 9.93, *SD* = 2.75)	*N* = 119, Age NR, recruited via Community setting	1. SCL-90-A[33]	Dyadic child-parent treatment.	Family-based
Poole et al. (2018),[34] Australia	RCT	Major DD, minor DD and dysthymic disorder. 12-18 (*m* = 15.2, *SD* = 1.4)	*N* = 64, Age *m* = 47.1, *SD* = 5.6, 92% female, recruited via community setting	1. DASS-A[31]<br>2. DASS-D[31]<br>3. DASS-S[31]	BEST MOOD Programme	Family-based
Racey et al. (2018),[35] UK	Mixed methods (feasibility)	Depression with or without comorbid GAD or PAD. 14-18 (*m* = 16.4, *SD* = 1.0)	*N* = 21, Age 36-53 (*m* = 47.8, *SD* = 5.0), 100% female - mothers, recruited via Child and Adolescent Mental Health Service	1. BDI-II[13]<br>2. RRS[36]<br>3. SCS[37]<br>4. MAAS[38]<br>5. EQDS[39]	Mindfulness-Based Cognitive Therapy	Mindfulness
Reigstad et al. (2022),[40] USA	Mixed-methods (feasibility)	Depression. 12-18 (*m* = 15.0, *SD* = 2.34)	*N* = 15, Age NR, 93% female - mothers, 7% fathers, recruited via outpatient clinic	1. SIPA[41]<br>2. SIPA-APRD[41]	Healthy Emotions and Relationships with Teens-A Guide for Parents	Attachment parenting
Salari et al. (2018),[42] Iran	RCT	GAD, separation anxiety, social phobia and specific phobia. 6-12 (*m* = 8.28, *SD* = 2.2)	*N* = 20, Age *m* = 34.8, SD = 5.0 , 86% female – mothers, 80-90% employed, recruited via outpatient clinic.	1. DASS-S[19]<br>2. GRAF[43]	Parent training components of FRIENDS for life	Group CBT
Waters et al. (2009),[44] Australia	RCT	GAD, SAD, specific phobia and social phobia. 4-8 (*m*= 6.8)	*N* = 49, mostly mothers, employment status: 66% mothers employed and 82% fathers	1. PSCS[45]<br>2. DASS-42-A[31]<br>3. DASS-42-D[31]	TAKE ACTION – Group cognitive behavioural therapy	Group CBT

Author, date, country	Study design	CYP diagnosis and age (years)	Participant characteristics and recruitment (where reported)	Parent outcome measures	Intervention name[a]	Intervention type
			employed, recruited via community setting	4. DASS-42-S[31]		

Notes: CYP; children and young people, RCT; randomised controlled trial, OCD; obsessive compulsive disorder, *m*; mean, QoLI; Quality of life inventory, *SD*; standard deviation, MCMI-III; Millon Clinical Multiaxial Inventory 3rd edition, MCMI-III; Millon Clinical Multiaxial Inventory – Compulsive subscale, RSQ-AvA; Relationship Scales Questionnaire – avoidant attachment subscale, RSQ-AnA; Relationship Scales Questionnaire – anxious attachment subscale, BEST; Behaviour Exchange Systems Training, ADHD; Attention deficit hyperactivity disorder, CES-D; Centre for Epidemiologic Studies-Depression Scale, CSQ; Caregiver Strain Questionnaire, DD; depressive disorder, UMDQ; Knowledge - Understanding Mood Disorders Questionnaire, EEAC+; The Expressed Emotion Adjective Checklist-Positive, EEAC-; The Expressed Emotion Adjective Checklist-Negative, BDI-II; Beck Depression Inventory II, PES-B; Parent Experiences Scale-Burden, PMS; Pearlin Mastery Scale, SPSI-R:L; Social Problem Solving Inventory-revised, Long Version, PSS; Perceived Stress Scale, DASS-A; Depression, Anxiety, Stress Scale-anxiety subscale, DASS-D; Depression, Anxiety, Stress Scale-depression subscale, SPWB; Scales of Psychological Wellbeing, MOSS-SSS; Medical Outcomes Study: Social Support Survey, MDD; major depressive disorder, GAD; generalised anxiety disorder, BP-II; bipolar II disorder, PSES; Parental Self-Efficacy Scale, BAS; Burden Assessment Scale, K6; Kessler Psychological Distress Scale, P-A:A; Parent-adolescent attachment, PRADAS; Parenting to Reduce Adolescent Depression and Anxiety Scale, TBQ-P; The Treatment Beliefs Questionnaire-Parent, CBT; cognitive behavioural therapy, NR; not reported, ADIS-IV; Anxiety Disorders Interview Schedule for DSM-IV, CBQ; Conflict behaviour questionnaire, SCL-90-A; Symptom Checklist-90-Anxiety, DASS-S; Depression, Anxiety, Stress Scale-stress subscale, RRS; Rumination Response Scale, PAD; phobic anxiety disorder, SCS; Self Compassion Scale, MAAS; Mindful Attention Awareness Scale, EQDS; The Experiences Questionnaire Decentring Subscale, SIPA; Stress Index for Parents of Adolescents, SIPA-APRD; Stress Index for Parents of Adolescents-Adolescent-Parent Relationship Domain, GRAF; Global Relational Assessment of Functioning, PSCS; Parents Sense of Competency Scale

[a]The naming convention for interventions was based on the emphasis of intervention contents. For example, interventions labelled as ‘group CBT’ was due to CBT being a predominant focus. If there were a range of family-based processes that were targeted, the labelling was therefore ‘family-based’.

Table 2 – Intervention details

Author, date, country	Intervention details	Mode of delivery	Frequency, duration and length of intervention	Summary of intervention components
Abedi and Vostanis (2010),[1] Iran	To increase quality of life across core areas of life satisfaction. Seeks to change core QoLT concepts, attitudes, skills, strengths and positive schemas to promote lasting life satisfaction and sense of contentment. Problem solving, with techniques e.g. increasing quality time with child, presented each session. Delivered as standalone parent support, via therapist.	Face-to-face.	Eight 90-minute sessions over 4 weeks.	Cognitive therapy techniques, "life management" skills.
Bertino et al (2013),[3] Australia	To increase family cohesion and reduce mental health symptoms in youth and parents. Psychoeducation pertaining to adolescent development including the need for individuation and increasing youth responsibility. Weekly family homework tasks and a whole-of-family approach was utilised to increase family cohesion. Delivered as Family therapy with interventions for parents, via therapist.	Face-to-face.	Eight sessions, over eight weeks (CYP were invited and encouraged to join the last four sessions).	Psychoeducation and parenting strategies, support for parents' emotional wellbeing, responsibility and shame.
Boxmeyer (2004),[6] USA	To reduce CYP functional impairment and improve family relationships. A variety of publicly-funded outpatient mental health treatments provided the following: family systems, electic, cognitive behavioural, psychodynamic and humanistic/other. Delivered as Family therapy with interventions for parents, via therapist.	Face-to-face.	157 sessions (*m* = 14.2, *SD* = 9.3) over a six-month period.	Varied support for parents including family systems, CBT, and eclectic approaches.
Fristad et al. (2003),[9] USA	To: i) increase parental knowledge of children's mood disorders and ii) improve family interaction. Parents and children attend their own break-out sessions that provides social support, information about mood symptoms and disorders and social skills building. Children's break-out sessions are more interactive and hands-on, consistent with the development needs of the population. Delivered as Family therapy with intervention for parents, via therapist.	Face-to-face.	Six Sessions.	Psychoeducation program, group discussion, workbooks, skills training, cognitive-behavioural approaches, problem solving.
Gerkensmeyer et al. (2013),[12] USA	To identify primary caregivers' depressive symptoms and feelings of burden and link them to problems in living. 7 steps: i) selecting and defining a problem, ii) establishing realistic and achievable goals for problem resolution and iii) generating multiple solution alternatives, iv) implementing decision-making guidelines, v) evaluating and choosing solutions, vi) implementing caregiver-selected solutions, and vii) evaluating outcomes from the previous week (except for the first week). Delivered as Standalone parent support via therapist.	Telephone and face-to-face.	Eight weekly 30-minute telephone sessions that followed a 1-hour face to face training session.	Cognitive-behavioural, problem solving.

Author, date, country	Intervention details	Mode of delivery	Frequency, duration and length of intervention	Summary of intervention components
Gleeson et al. (2017),[17] Australia	To reduce stress in caregivers. Online therapy content combined with purpose built Facebook-esque online social networking, and peer and expert moderation. Facilitation of skills practice or behavioural experiments were provided in line with identified personal strengths and needs. Behavioural goal setting was also provided and others could join to support them in a "team up" challenge. Delivered as standalone parent support.	Digital therapy, online platform	Facilitators had weekly supervision meetings with clinical psychologists with 24 h per day access to online platform.	Self-care support, psychoeducation, communication strategies, coping strategies. Peer-support.
Khor et al. (2021),[22] Australia	To target modifiable parenting factors associated with adolescent anxiety and/or depression. Composed of two parts: i) web-based intervention and ii) therapist-facilitated coaching. Nine weekly-web-based modules are automatically recommended such as conflict management at home and problem solving. Therapist-facilitated coaching included psychoeducation about adolescent anxiety and depression and up to 12 telehealth sessions, one for each online module (with the exception of Good Health Habits). Delivered as Standalone parent support, via therapist and peers.	Online (web-based and teleconferences)	15-25 minute for each online module and 45-60 minute telehealth weekly sessions. 1 hour per fortnight of clinical supervision and weekly peer supervision.	Psychoeducation, problem solving, teaching parenting strategies, good health management e.g. sleep, diet, coping with own anxiety.
MacPherson et al. (2016),[28] USA	To improve understanding and management of mood disorders and belief that child will improve through treatment. Family workbooks and parent only groups. Material re: family interactions and parents' own unhelpful cognitive patterns, and strategies to support child emotional regulation. Family projects set. Delivered as group, via therapist.	Face-to-face.	Eight 90-minute sessions.	Psychoeducation, family therapy, CBT, strategies avoid dysfunctional family cycles.
O'Brien et al. (2007),[30] Ireland	To provide psychoeducation to reduce parents' own anxiety, alongside child's treatment. Delivered by mental health professionals. Manual and workbook based-CBT intervention that involved psychoeducation that focused on learning mind-body, cognitive and emotional awareness and the development of cognitive and behavioural strategies for dealing with anxiety.	Face-to-face.	Three, 90-minute sessions.	Psychoeducation regarding: parental anxiety management.
Pina (2005),[32] USA	To enhance parent-child communication, problem solving and reduce parent anxiety symptoms. Sessions with handouts and homework. Sessions 4-6 involved targeting specific parent-child relational processes. Delivered as Family therapy with interventions for parents, via therapist.	Face-to-face.	Twelve, 80 minute sessions.	CBT for anxiety, problem solving, communication skills.
Poole et al. (2018),[34] Australia	To optimise youth and family mental Improve parent self-care, stress management strategies, promoting parental confidence, family connectedness and enhancing family communication. Delivered as Family therapy with interventions for parents, via therapist.	Face-to-face.	Eight, 2-hour sessions.	Family therapy. Behavioural activation. Psychoeducation.

**Author, date, country**	**Intervention details**	**Mode of delivery**	**Frequency, duration and length of intervention**	**Summary of intervention components**
				Coping and management strategies.
Racey et al. (2018),[35] UK	To develop mindfulness skills for parents and young people. Sessions delivered via therapist involved a series of explanations, focused exercises and group meditation practices followed by small and large group discussions that: i) encouraged young people's awareness of inattention and to increase ability to direct their attention to the experience of the present moment and ii) increase young people's awareness of habitual patterns of reactivity and associated judgements and behaviours. Participants were also encouraged to reinforce all this with formal practice at home between sessions.	Face-to-face.	Eight sessions.	Psychoeducation. Mindfulness.
Reigstad et al. (2022),[40] USA	To address attachment parenting, including parent responses to emotions. Skills to support secure attachment. Discussion of situations parents are having difficulty with and their experience of parenting. Delivered as standalone parent support, via psychologist.	Face-to-face.	Eight sessions - manualised.	Psychoeducation. Attachment focused strategies. Parenting skills, including communication skills.
Salari et al. (2018),[42] Iran	To help parents' recognise and manage their own anxiety and support child to also. Delivered as parent intervention within CYP CBT, via therapist.	Face-to-face.	Six weekly, two-hour sessions.	Psychoeducation, stress management techniques.
Waters et al. (2009),[44] Australia	To provide strategies to managing child anxiety and improving the parent-child communication and relationship, and parental coping. Workbooks and homework. Delivered as Standalone parent support, via therapist.	Face-to-face.	Ten weekly, 1-hour sessions, with booster session 8 weeks after final session.	Psychoeducation. Coping strategies. Communication strategies.

1. Abedi MR and Vostanis P. Evaluation of quality of life therapy for parents of children with obsessive-compulsive disorders in Iran. *Eur Child Adolesc Psychiatry* 2010; 19: 605-613. 2010/02/17. DOI: 10.1007/s00787-010-0098-4.
2. Frisch MB. Use of the QOLI® or Quality of Life InventoryTM in Quality of Life Therapy and Assessment. *The use of psychological testing for treatment planning and outcomes assessment: Instruments for adults, Volume 3, 3rd ed*. Mahwah, NJ, US: Lawrence Erlbaum Associates Publishers, 2004, pp.749-797.
3. Bertino MD, Richens K, Knight T, et al. Reducing parental anxiety using a family based intervention for youth mental health: A randomized controlled trial. *Open Journal of Psychiatry* 2013; Vol.03No.01: 13. DOI: 10.4236/ojpsych.2013.31A013.
4. Millon T, Davis RD and Millon C. *MCMI-III Manual*. Minneapolis, Minn.: National Computer Systems Pearson, 1997.
5. Griffin DW and Bartholomew K. The metaphysics of measurement: The case of adult attachment. In: Bartholomew K and Perlman D (eds) *Attachment Processes in Adulthood, Advances in Personal Relationships*. London: Jessica Kingsley Publishers, 1994, pp.17-52.
6. Boxmeyer CL. *Parent and Family Outcomes of Community-Based Mental Health Treatment for Adolescents*. San Diego State University, 2004.
7. Radloff LS. The CES-D scale: A self-report depression scale for research in the general population. *Applied psychological measurement* 1977; 1: 385-401.
8. Brannan AM, Heflinger CA and Bickman L. The Caregiver Strain Questionnaire:Measuring the Impact on the Family of Living with a Child with Serious Emotional Disturbance. *Journal of Emotional and Behavioral Disorders* 1997; 5: 212-222. DOI: 10.1177/106342669700500404.
9. Fristad MA, Goldberg-Arnold JS and Gavazzi SM. Multi-family psychoeducation groups in the treatment of children with mood disorders. *J Marital Fam Ther* 2003; 29: 491-504. 2003/11/05. DOI: 10.1111/j.1752-0606.2003.tb01691.x.
10. Gavazzi SM, Fristad MA and Law JC. The Understanding Mood Disorders Questionnaire. *Psychol Rep* 1997; 81: 172-174. DOI: 10.2466/pr0.1997.81.1.172.
11. Friedmann MS and Goldstein MJ. Relatives' awareness of their own expressed emotion as measured by a self-report adjective checklist. *Fam Process* 1993; 32: 459-471. 1993/12/01. DOI: 10.1111/j.1545-5300.1993.00459.x.
12. Gerkensmeyer JE, Johnson CS, Scott EL, et al. Problem-solving intervention for caregivers of children with mental health problems. *Arch Psychiatr Nurs* 2013; 27: 112-120. 2013/05/28. DOI: 10.1016/j.apnu.2013.01.004.
13. Beck AT, Steer RA and Brown GK. Beck depression inventory-II. *San Antonio* 1996; 78: 490-498.
14. Gerkensmeyer JE, Perkins SM, Scott EL, et al. Depressive Symptoms Among Primary Caregivers of Children With Mental Health Needs: Mediating and Moderating Variables. *Archives of Psychiatric Nursing* 2008; 22: 135-146. DOI: https://doi.org/10.1016/j.apnu.2007.06.016.
15. Pearlin LI and Schooler C. The structure of coping. *J Health Soc Behav* 1978; 19: 2-21. 1978/03/01.
16. D'Zurilla TJ and Nezu AM. Development and preliminary evaluation of the Social Problem-Solving Inventory. American Psychological Association, 1990, p. 156-163.
17. Gleeson J, Lederman R, Koval P, et al. Moderated Online Social Therapy: A Model for Reducing Stress in Carers of Young People Diagnosed with Mental Health Disorders. *Front Psychol* 2017; 8: 485. 2017/04/20. DOI: 10.3389/fpsyg.2017.00485.
18. Cohen S, Kamarck T and Mermelstein R. Perceived stress scale. *Measuring stress: A guide for health and social scientists* 1994; 10: 1-2.
19. Brown TA, Chorpita BF, Korotitsch W, et al. Psychometric properties of the Depression Anxiety Stress Scales (DASS) in clinical samples. *Behaviour research and therapy* 1997; 35: 79-89.
20. Ryff CD and Keyes CL. The structure of psychological well-being revisited. *J Pers Soc Psychol* 1995; 69: 719-727. DOI: 10.1037//0022-3514.69.4.719.

21. Sherbourne CD and Stewart AL. The MOS social support survey. *Soc Sci Med* 1991; 32: 705-714. 1991/01/01. DOI: 10.1016/0277-9536(91)90150-b.
22. Khor SPH, Fulgoni CM, Lewis D, et al. Short-term outcomes of the Therapist-assisted Online Parenting Strategies intervention for parents of adolescents treated for anxiety and/or depression: A single-arm double-baseline trial. *Aust N Z J Psychiatry* 2022; 56: 695-708. 2021/07/08. DOI: 10.1177/00048674211025695.
23. Nicolas CC, Jorm AF, Cardamone-Breen MC, et al. Parental Self-Efficacy for Reducing the Risk of Adolescent Depression and Anxiety: Scale Development and Validation. *Journal of research on adolescence : the official journal of the Society for Research on Adolescence* 2020; 30: 249-265. 20190627. DOI: 10.1111/jora.12521.
24. Reinhard SC, Gubman GD, Horwitz AV, et al. Burden assessment scale for families of the seriously mentally ill. *Evaluation and Program Planning* 1994; 17: 261-269. DOI: https://doi.org/10.1016/0149-7189(94)90004-3.
25. Kessler RC, Andrews G, Colpe LJ, et al. Short screening scales to monitor population prevalences and trends in non-specific psychological distress. *Psychol Med* 2002; 32: 959-976. DOI: 10.1017/s0033291702006074.
26. McElhaney KB, Porter MR, Thompson LW, et al. Apples and Oranges: Divergent Meanings of Parents' and Adolescents' Perceptions of Parental Influence. *J Early Adolesc* 2008; 28: 206-229. 2008/06/20. DOI: 10.1177/0272431607312768.
27. Cardamone-Breen MC, Jorm AF, Lawrence KA, et al. The Parenting to Reduce Adolescent Depression and Anxiety Scale: Assessing parental concordance with parenting guidelines for the prevention of adolescent depression and anxiety disorders. *PeerJ* 2017; 5: e3825. 2017/09/28. DOI: 10.7717/peerj.3825.
28. MacPherson HA, Mackinaw-Koons B, Leffler JM, et al. Pilot effectiveness evaluation of community-based multi-family psychoeducational psychotherapy for childhood mood disorders. *Couple and Family Psychology: Research and Practice* 2016; 5: 43-59. DOI: 10.1037/cfp0000055.
29. Davidson KH and Fristad MA. The Treatment Beliefs Questionnaire (TBQ): An instrument to assess beliefs about children's mood disorders and concomitant treatment needs. *Psychological Services* 2006; 3: 1-15. DOI: 10.1037/1541-1559.3.1.1.
30. O'Brien F, Olden N, Migone M, et al. Group cognitive behavioural therapy for children with anxiety disorder - an evaluation of the 'Friends for Youth' programme. *Ir J Psychol Med* 2007; 24: 5-12. 2007/03/01. DOI: 10.1017/s0790966700010065.
31. Lovibond PF and Lovibond SH. The structure of negative emotional states: Comparison of the Depression Anxiety Stress Scales (DASS) with the Beck Depression and Anxiety Inventories. *Behaviour research and therapy* 1995; 33: 335-343.
32. Pina AA. *Child Parent Dyadic Treatment For Anxiety Disorders In Youths*. Florida International University, 2005.
33. Derogatis LR and Unger R. Symptom Checklist-90-Revised. *The Corsini Encyclopedia of Psychology*. John Wiley & Sons, Inc., 2010.
34. Poole LA, Knight T, Toumbourou JW, et al. A Randomized Controlled Trial of the Impact of a Family-Based Adolescent Depression Intervention on both Youth and Parent Mental Health Outcomes. *J Abnorm Child Psychol* 2018; 46: 169-181. 2017/04/05. DOI: 10.1007/s10802-017-0292-7.
35. Racey DN, Fox J, Berry VL, et al. Mindfulness-Based Cognitive Therapy for Young People and Their Carers: a Mixed-Method Feasibility Study. *Mindfulness (N Y)* 2018; 9: 1063-1075. 2018/08/14. DOI: 10.1007/s12671-017-0842-7.
36. Treynor W, Gonzalez R and Nolen-Hoeksema S. Rumination reconsidered: A psychometric analysis. *Cognitive therapy and research* 2003; 27: 247-259.
37. Neff KD. The self-compassion scale is a valid and theoretically coherent measure of self-compassion. *Mindfulness* 2016; 7: 264-274.
38. Brown KW and Ryan RM. The benefits of being present: Mindfulness and its role in psychological well-being. *Journal of Personality and Social Psychology* 2003; 84: 822-848. doi:10.1037/0022-3514.84.4.822. DOI: 10.1037/0022-3514.84.4.822.
39. Fresco DM, Moore MT, van Dulmen MH, et al. Initial psychometric properties of the experiences questionnaire: validation of a self-report measure of decentering. *Behav Ther* 2007; 38: 234-246. 2007/08/19. DOI: 10.1016/j.beth.2006.08.003.
40. Reigstad KM, Gunlicks-Stoessel ML, Westervelt A, et al. Healthy emotions and relationships with teens-a guide for parents: An intervention development study of a parent-based intervention for adolescents with depression. *Clin Child Psychol Psychiatry* 2022; 27: 586-597. 2022/03/26. DOI: 10.1177/13591045221078416.
41. Sheras P, Abidin R and Konold T. Stress index for parents of adolescents: Professional manual. Odessa, FL: Psychological Assessment Resources. Inc, 1998.

42. Salari E, Shahrivar Z, Mahmoudi-Gharaei J, et al. Parent-only Group Cognitive Behavioral Intervention for Children with Anxiety Disorders: A Control Group Study. *J Can Acad Child Adolesc Psychiatry* 2018; 27: 130-136. 2018/04/18.
43. Group For The Advancement Of Psychiatry Committee On The F. Global Assessment of Relational Functioning Scale (GARF): I. Background and Rationale. *Family Process* 1996; 35: 155-172. DOI: https://doi.org/10.1111/j.1545-5300.1996.00155.x.
44. Waters AM, Ford LA, Wharton TA, et al. Cognitive-behavioural therapy for young children with anxiety disorders: Comparison of a Child + Parent condition versus a Parent Only condition. *Behav Res Ther* 2009; 47: 654-662. 2009/05/22. DOI: 10.1016/j.brat.2009.04.008.
45. Johnston C and Mash EJ. A measure of parenting satisfaction and efficacy. *Journal of clinical child psychology* 1989; 18: 167-175.
