# Supplementary material for: Systematic review and meta-analysis of interventions to improve outcomes for parents or carers of children with anxiety and/or depression
Source: BMJ Ment Health. 2024 Sep 25;27(1):e301218. doi: 10.1136/bmjment-2024-301218 (PMC11425941; doi:10.1136/bmjment-2024-301218)
Supplement: online supplemental material 3 [file bmjment-27-1-s005.pdf]

	Component A: Selection Bias			Component B: Study Design				Component C: Confounders			Component D: Blinding			Component E: Data Collection Methods			Component F: Withdrawals and Drop outs			Component G: Intervention Integrity			Component H: Analyses				
	Are the individuals selected to participate in the stud likely to be representative of the target population?	What percentage of selected individuals agreed to participate?	Rate This Section (1 = Strong , 2 = Moderate , 3 = Weak)	Indicate the study design	Was the study described as randomised? If No, go t component C	If Yes, was the method of randomisation described?	Rate This Section (1 = Strong , 2 = Moderate , 3 = Weak)	Were there important differences between groups prior to the intervention?	If yes, indicate the percentage of relevant confounders that were controlled (either in the desigr (e.g. stratification, matching) or analysis)?	Rate This Section (1 = Strong , 2 = Moderate , 3 = Weak)	Was (were) the outcome assessor(s) aware of the intervention or exposure status of participants?	Were the study participants aware of the research question?	Rate This Section (1 = Strong , 2 = Moderate , 3 = Weak)	Were data collection tools shown to be valid?	Were data collection tools shown to be reliable?	Rate This Section (1 = Strong , 2 = Moderate , 3 = Weak)	Were withdrawals and drop-outs reported in terms of numbers and/or reasons per group?	Indicate the percentage of participants completing the study. (If the percentage differs by groups, recorc the lowest)	Rate This Section (1 = Strong , 2 = Moderate , 3 = Weak)	What percentage of participants received the allocated intervention or exposure of interest?	Was the consistency of the intervention measured?	Is it likely that subjects received an unintended intervention (Contamination or Co-Intervention) that may influence the results?	Indicate the unit of allocation (SELECT ONE)	Indicate the unit of analysis (SELECT ONE)	Are the statistical methods appropriate for the study design?	Is the analysis performed by the intervention allocation status (i.e. intention to treat) rather than th actual intervention received?	**Global Rating for this paper (1 = Strong (No Weak Ratings) 2= Moderate (One Weak Rating), 3 = Weak (Two or More WEAK ratings))**
Abedi and Vostanis (2010)	2 Somewhat likely	1 80-100% agreement	2 Moderate	Randomised Control Trial	Yes	Yes	1 Strong	2 No	N/A	1 Strong	1Yes	1Yes	3 Weak	1Yes	1Yes	1 Strong	1Yes	1 80-100%	1 Strong	1 80-100%	1Yes	2 No	Individual	Individual	1Yes	2 No	2 Moderate
Bertino et al (2013)	2 Somewhat likely	2 60 -79% agreement	2 Moderate	Randomised Control Trial	Yes	Yes	1 Strong	2 No	N/A	1 Strong	1Yes	2 No	2 Moderate	1Yes	1Yes	1 Strong	1Yes	Less than 60%	3 Weak	2 60 -79%	1Yes	2 No	Individual	Individual	1Yes	1Yes	2 Moderate
Boxmeyer (2004)	2 Somewhat likely	2 60-79% agreement	2 Moderate	Cohort (one group pre + pos	No	N/A	2 Moderate	2 No	N/A	1 Strong	1Yes	1 yes	3 Weak	1Yes	1Yes	1 Strong	1Yes	1 80-100%	1 Strong	1 80-100%	1Yes	2 No	Individual	Individual	1Yes	2 No	2 Moderate
Fristad et al. (2003)	2 Somewhat likely	1 80-100% agreement	2 Moderate	Randomised Control Trial	Yes	Yes	1 Strong	2 No	N/A	1 Strong	1Yes	1Yes	3 Weak	1Yes	1Yes	1 Strong	1Yes	1 80-100%	1 Strong	Less than 60%	1Yes	2 No	Individual	Individual	1 yes	2 No	2 Moderate
Gerkensmeyer et al. (2013)	2 Somewhat likely	1 80-100% agreement	2 Moderate	Randomised Control Trial	Yes	Yes	1 Strong	2 No	N/A	1 Strong	1Yes	1Yes	3 Weak	1Yes	1Yes	1 Strong	1Yes	1 80-100%	1 Strong	1 80- 100%	1Yes	2 No	Individual	Individual	1 yes	2 No	2 Moderate
Gleeson et al. (2017)	2 Somewhat likely	1 80-100 % agreement	2 Moderate	Other (Feasability)	No	No	3 Weak	2 No	N/A	1 Strong	1Yes	1Yes	3 Weak	1Yes	1Yes	1 Strong	2 No	1 80-100%	1 Strong	1 80-100%	1Yes	2 No	Individual	Individual	1Yes	2 No	3 Weak
Khor et al. (2021)	2 Somewhat likely	1 80-100% agreement	2 Moderate	6 interrupted time series	No	No	2 Moderate	2 No	N/A	1 Strong	1Yes	3 Can't tell	3 Weak	1Yes	1Yes	1 Strong	1Yes	1 80-100%	1 Strong	Less than 60%	1Yes	2 No	Individual	Individual	1Yes	2 No	2 Moderate
MacPherson et al. (2016)	2 Somewhat likely	5 Can't tell	2 Moderate	5 cohort (one group pre + post (Before and After))	No	No	2 Moderate	2 No	N/A	1 Strong	1Yes	1Yes	3 Weak	1Yes	1Yes	1 Strong	2 No	2 60-79%	2 Moderate	1 80 - 100%	3 Can't Tell	2 No	Individual	Individual	1Yes	1Yes	2 Moderate
O'Brien et al. (2007)	2 Somewhat likely	2 60-79% agreement	2 Moderate	Randomised Control Trial	Yes	Yes	1 Strong	3 Can't tell	N/A	1 Strong	2 No	1Yes	2 Moderate	1Yes	1Yes	1 Strong	1Yes	1 80-100%	1 Strong	1 80 - 100%	1Yes	2 No	Individual	Individual	1Yes	2 No	1 Strong
Pina (2005)	2 Somewhat likely	1 80-100% agreement	2 Moderate	Randomised controlled tria	Yes	Yes	1 Strong	2 No	N/A	1 Strong	2 No	1Yes	2 Moderate	1Yes	1Yes	1 Strong	1Yes	2 60-79%	2 Moderate	1 80 - 100%	1Yes	2 No	Individual	Individual	1Yes	2 No	1 Strong
Poole et al. (2018)	2 Somewhat likely	1 80-100% agreement	2 Moderate	Randomised controlled tria	Yes	Yes	1 Strong	2 No	N/A	1 Strong	2 No	2 No	1 Strong	1Yes	1Yes	1 Strong	1Yes	2 60-79%	2 Moderate	1 80 - 100%	2 No	2 No	Individual	Individual	1Yes	1Yes	1 Strong
Racey et al. (2018)	2 Somewhat likely	1 80-100% agreement	2 Moderate	ne group pre + post (Before	No	N/A	2 Moderate	2 No	N/A	1 Strong	1Yes	1Yes	3 Weak	1Yes	1Yes	1 Strong	1Yes	1 80-100%	1 Strong	1 80 - 100%	3 Can't Tell	2 No	Individual	Individual	1Yes	2 No	2 Moderate
Reigstad et al. (2022	2 Somewhat likely	1 80-100% agreement	2 Moderate	ne group pre + post (Before	No	N/A	2 Moderate	2 No	N/A	1 Strong	1Yes	3 Can't tell	3 Weak	1Yes	1Yes	1 Strong	1Yes	1	1 Strong	1 80 - 100%	1Yes	2 No	Individual	Individual	1Yes	1Yes	2 Moderate
Salari et al. (2018)	3 Not Likely	1 80-100% agreement	3 Weak	Randomised controlled tria	Yes	Yes	1 Strong	2 No	N/A	1 Strong	1Yes	1Yes	3 Weak	1Yes	1Yes	1 Strong	1Yes	2 60-79%	2 Moderate	1 80 - 100%	1Yes	2 No	Individual	Individual	1Yes	2 No	3 Weak
Waters et al. (2009)	2 Somewhat likely	1 80-100% agreement	2 Moderate	Randomised controlled tria	Yes	Yes	1 Strong	2 No	N/A	1 Strong	2 No	1Yes	2 Moderate	1Yes	1Yes	1 Strong	2 No	2 60-79%	2 Moderate	1 80 - 100%	1Yes	2 No	Individual	Individual	1Yes	1Yes	1 Strong
